# Defining short linear motif binding determinants by phage display‐based deep mutational scanning

**DOI:** 10.1002/pro.70174

**Published:** 2025-05-24

**Authors:** Caroline Benz, Lars Maassen, Leandro Simonetti, Filip Mihalic, Richard Lindqvist, Ifigenia Tsitsa, Aimiliani Konstantinou, Per Jemth, Anna K. Överby, Norman E. Davey, Ylva Ivarsson

**Affiliations:** ^1^ Department of Chemistry – BMC Uppsala University Uppsala Sweden; ^2^ Department of Medical Biochemistry and Microbiology Uppsala University, BMC Uppsala Sweden; ^3^ Department of Clinical Microbiology Umeå University Umeå Sweden; ^4^ Laboratory for Molecular Infection Medicine Sweden (MIMS) Umeå University Umeå Sweden; ^5^ Division of Cancer Biology The Institute of Cancer Research London UK

**Keywords:** deep mutational scanning, NSP9, peptide‐phage display, SARS‐CoV‐2, short linear motif

## Abstract

Deep mutational scanning (DMS) has emerged as a powerful approach for evaluating the effects of mutations on binding or function. Here, we developed a DMS by phage display protocol to define the specificity determinants of short linear motifs (SLiMs) binding to peptide‐binding domains. We first designed a benchmarking DMS library to evaluate the performance of the approach on well‐known ligands for 11 different peptide‐binding domains, including the talin‐1 PTB domain, the G3BP1 NTF2 domain, and the MDM2 SWIB domain. Comparison with a set of reference motifs from the eukaryotic linear motif (ELM) database confirmed that the DMS by phage display analysis correctly identifies known motif binding determinants and provides novel insights into specificity determinants, including defining a non‐canonical talin‐1 PTB binding motif with a putative extended conformation. A second DMS library was designed, aiming to provide information on the binding determinants for 19 SLiM‐based interactions between human and SARS‐CoV‐2 proteins. The analysis confirmed the affinity determining residues of viral peptides binding to host proteins and refined the consensus motifs in human peptides binding to five domains from SARS‐CoV‐2 proteins, including the non‐structural protein (NSP) 9. The DMS analysis further pinpointed mutations that increased the affinity of ligands for NSP3 and NSP9. An affinity‐improved cell‐permeable NSP9‐binding peptide was found to exert stronger antiviral effects than the wild‐type peptide. Our study demonstrates that DMS by phage display can efficiently be multiplexed and applied to refine binding determinants and shows how the results can guide peptide‐engineering efforts.

## 1 INTRODUCTION

Short linear motifs (SLiMs) are compact protein–protein interaction modules typically found in the intrinsically disordered regions (IDRs) of the proteome (Tompa et al., 2014). SLiM‐based interactions play a crucial role in several important cellular processes such as signal transduction, enzyme recruitment, and protein localization. A general picture of SLiM‐based interactions has emerged where a limited set of 3–4 key residues serve as the main specificity and affinity determinants, and motif‐flanking regions modulate binding (Holehouse & Kragelund, 2024; Kumar et al., 2024; Mihalic et al., 2024). Disease‐associated mutations in the IDRs have been found to both break and make SLiM‐based interactions (Kliche et al., 2024; Meszaros et al., 2017; Rrustemi et al., 2024). Furthermore, viruses exploit SLiM‐based interactions to both hijack and deregulate the host cell machinery. Viral SLiMs bind to host proteins (Davey et al., 2011; Mihalic, Simonetti, et al., 2023), and folded viral proteins bind to host SLiMs (Madhu et al., 2022; Mihalic, Benz, et al., 2023). Both scenarios offer the possibility to inhibit viral infection by blocking the SLiM‐binding pockets (Kruse et al., 2021; Mihalic Benz, et al., 2023; Simonetti et al., 2023). Finding and optimizing SLiM‐based interactions between viral and human proteins thus offer potential strategies to develop antivirals.

Defining a SLiM requires both the identification of the binding peptide region and pinpointing the key residues that confer affinity and specificity. Both can be accomplished by methods such as proteomic peptide phage display (ProP‐PD) (Benz et al., 2022). However, in some cases, such analysis returns only limited sets of peptide ligands, preventing the identification of shared consensus motifs. Additional experiments, such as alanine scanning peptide arrays, point mutations, or structural analysis, are subsequently required to define the key residues in these peptides. Moreover, most approaches used assume that the binding determinants of binding‐enriched peptides conform to one dominating motif consensus. Information on motif variations and more subtle contributions of motif‐flanking residues are rarely captured by these approaches. During the last decade, deep mutational scanning (DMS) has emerged as a powerful variant of saturation mutagenesis approaches to define the effects of all possible mutations on binding (Fowler & Fields, 2014). DMS can be used to evaluate how the binding between a protein and a given peptide is affected by replacing each amino acid in a peptide sequence with all other amino acids in a saturation mutagenesis library where the phenotype is linked to the genotype (e.g., by yeast display). Deep sequencing of the library before and after sorting/selection determines the relative abundance, and thereby the relative binding to the bait, of each sequence (Claussnitzer et al., 2024; Davey et al., 2023). DMS has, for example, been utilized to explore motif‐mediated interactions of PDZ (PSD‐95, Discs‐large, ZO‐1) and SH3 (Src Homology 3) domains (Faure et al., 2022), to characterize the LxxP docking motif for the yeast cyclin Cln2 (Bandyopadhyay et al., 2020), to investigate the peptide binding of TRAF domains (Foight & Keating, 2016), and to map antibody epitopes (Garrett et al., 2020).

**FIGURE 1:**
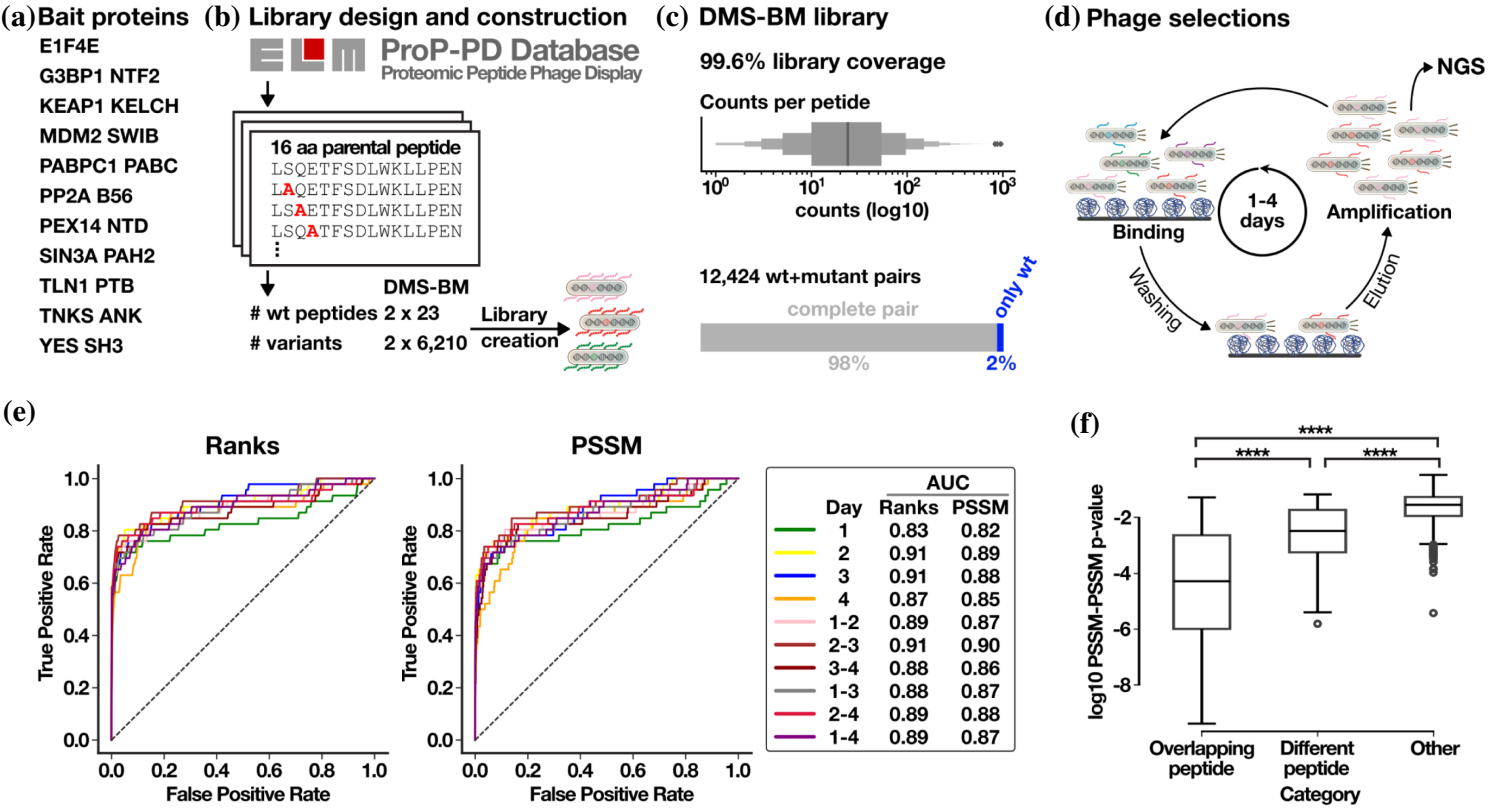
Overview of the benchmarking DMS analysis. (a) Bait protein domains selected for developing the DMS by phage protocol. (b) Schematic of the design of the DMS‐BM library. Overlapping parental peptides were selected based on data reported in the ELM database or in the ProP‐PD portal and used to generate the DMS phage library. (c) Coverage of the DMS‐BM phage display library. The read count distribution of all peptides found by input library sequencing is shown together with the percentage of wt/mutant pairs confirmed by NGS analysis. For 2% of the pairs, the mutant peptides were missing. (d) Schematic of the selections against the DMS‐BM library. The binding enriched phage pools were analyzed by NGS. (e) Receiver operating characteristic (ROC) analysis benchmarking the DMS by phage display analysis using Position‐Specific Scoring Matrices (PSSMs) created from the DMS‐BM selections, compared to PSSMs generated from peptide instances curated in the ELM database. Two scoring methods are compared: ranks and *p*‐values. Ranks refer to the ordered comparison scores from PSSM‐PSSM matching, where the rank of the expected ELM class relative to other classes is used for ROC analysis. *p*‐values correspond to the statistical significance of each PSSM‐PSSM comparison, quantifying the likelihood of observing the match between the expected ELM class and the DMS‐BM‐derived PSSMs by chance. Both methods assess the specificity and sensitivity of the predicted matches. (f) Comparison of the similarities of PSSMs generated based on results from overlapping peptides, from different peptides designed for the same bait, and from PSSMs for unrelated baits. **** indicates *p* < 0.0001.

In this study, we developed a peptide‐phage display‐based DMS protocol for parallel analysis of distinct SLiM‐based interactions using multiplexed phage libraries designed to contain multiple peptide saturation mutagenesis sub‐libraries. The method combines a designed oligonucleotide library, M13 peptide‐phage display, and next‐generation sequencing (NGS) (Figure 1). We first optimized the analysis pipeline for the phage display‐based DMS by benchmarking the analysis using a set of well‐studied SLiM‐based interactions (Benz et al., 2022; Kumar et al., 2024) and then applied the analysis to a set of poorly characterized interactions involving peptides or folded domains from the severe acute respiratory syndrome coronavirus‐2 (SARS‐CoV‐2) proteins (Kruse et al., 2021; Mihalic Benz, et al., 2023). We find that the DMS by phage display approach can be easily multiplexed by combining several saturation mutagenesis sub‐libraries into one larger phage display library, which can be used to determine binding specificities, pinpoint mutations that increase or decrease binding affinity, and provide directions for how to optimize the affinity of a given interaction. We demonstrate the utility of the strategy for engineering purposes by optimizing an antiviral peptide inhibitor binding to the nonstructural protein 9 (NSP9) from SARS‐CoV‐2.

## 2 RESULTS

### 2.1 Design and construction of the DMS by phage benchmarking library

We designed a DMS benchmarking (DMS‐BM) library (Table S1) to explore the effect of mutation on peptide binding to eleven human bait protein domains (Figure 1a), with distinct binding specificities (Table 1). Twenty‐three peptide ligands were retrieved from the Eukaryotic Linear Motif (ELM) database (Kumar et al., 2024) or from the ProP‐PD portal (Kliche et al., 2023) (Figure 1b). The design included well‐studied interactions such as the p53 degron peptide binding to the SWIB domain of the E3 ubiquitin‐protein ligase Mdm2 (MDM2) (Benz et al., 2022), and the cell division cycle‐associated protein 2 (CDCA2) LxxIxE motif‐containing peptide that binds to the B56 family protein phosphatase 2A (PP2A) regulatory subunit (Hertz et al., 2016). Furthermore, we included peptides from USP10 and CAPRIN1 that both bind to the NTF2‐like domain of G3BP1 but have distinct binding motifs (Schulte et al., 2023; Song et al., 2022). Also, two distinct ligands of the talin‐1 phosphotyrosine binding (TLN1 PTB) domain were included (Benz et al., 2022). Each SLiM was tiled with two overlapping parental peptides shifted by two amino acids (14 amino acid overlap). Fifteen of the positions in each peptide were subjected to in silico saturation mutagenesis (excluding cysteines for technical reasons), including the overlapping regions of the peptide pairs, resulting in 12,466 peptides (Figure 1b). The mutant peptide pool design was translated to oligonucleotides which were synthesized and genetically fused to the major coat protein P8 for multivalent display on the M13 phage. The sequence coverage of the constructed phage library was found to be 99.6%, with a balanced sequence representation (Figure 1c).

**TABLE 1:** Overview of domains and peptides used for the design of the DMS‐BM library, together with indication of the outcome of the analysis as evaluated by the completeness scores for the two overlapping peptides used.

Domain	Peptide gene name	Peptide sequences	Completeness of DMS results for peptide 1/peptide 2
EIF4E_1–217_	EIF4EBP1	_50−_ *TR*II**Y**DRKF**LM**ECRNS*PV*_−67_	0.55/0.46
	EIF4G1	_606−_ *LE*EKKR**Y**DREF**LL**GFQ*FI*_−623_	0.84/0.58
G3BP1 NFT2_1–139_	CAPRIN1	_362−_ *LM*AQMQGP**YNFI**QDSM*LD*_−379_	0.32/0.87
	USP10	_3−_ *LH*SPQ**Y**I**FGDF**SPDEF*NQ*_−20_	0.82/0.83
KEAP1 KELCH_321–609_	NFE2L1	_226−_ *RN*LLV**D**G**ETGE**SFPAQ*VP*_−243_	0.82/0.81
	SQSTM1	_342−_ *SS*KEV**D**P**STGE**LQSLQ*MP*_−359_	0.70/0.72
MDM2 SWIB_17–125_	KIAA1671	_600−_ *TP*EDDRS**F**QTV**W**AT**V**F*EH*_−617_	0.86/0.71
	RNF115	_67−_ *TT*TH**F**AEL**W**GH**L**DHTM*FF*_−84_	0.85/0.83
	TP53	_14−_ *LS*QET**F**SDL**W**KL**L**PEN*NV*_−31_	0.58/0.73
PABPC1 PABC_544–623_	ATXN2	_911−_ *KS*T**LNP**N**A**KE**F**N**P**RSF*SQ*_−928_	0.63/0.78
	PAIP1	_124−_ *LM*SK**LSV**N**A**PE**F**Y**P**SG*YS*_−141_	0.78/0.85
PP2A B56_1–486_	AXIN1	_235−_ *SG*YLPT**L**N**E**D**E**EWKCD*QD*_−252_	0.75/0.81
	CDCA2	_586−_ *KK*PL**L**SP**I**P**E**LPEVPE*MT*_−603_	0.74/0.84
PEX14 Pex14_16–84_	PEX5	_108−_ *GV*ADLALSEN**W**AQE**F**L*AA*_−125_	0.70/0.86
		_238−_ *AQ*AEQ**W**AAE**F**IQQQGT*SD*_−255_	0.74/0.89
SIN3A PAH2_295–383_	KLF9	_4−_ *AA*YMD**F**V**AA**Q**CLV**S**I**S*NR*_−21_	0.60/0.29
	MXI1	_4−_ *VK*MINVQRLLE**AA**EF**L** *ER*_−21_	0.65/0.84
TLN1 PTB_309–401_	PIP5K1C	_640−_ *FP*TDERS**W**VY**S**P**L**H*YS*_−657_	0.89/0.96
	TPTE2	_92−_ *LA*DLIFTD**S**K**L**YIPLE*YR*_−109_	0.75/0.69
TNKS ANK_174–649_	AMOTL2	_64−_ *QV*LQQAT**R**QE**P**Q**G**QEH*QG*_−81_	0.87/0.85
	SH3BP2	_408−_ *PQ*LPHLQ**R**SP**PDG**QSF*RS*_−425_	0.86/0.88
YES SH3_90–152_	BCAR1	_627−_ *DK*TSSIQS**R**PL**P**SP**P**K*FT*_−644_	0.86/0.87
	CBL	_538−_ *TL* **R**DL**P**PP**P**PPDRPYS*VG*_−555_	0.94/0.96

*Note*: Italic indicates residues that are found only in one of the two overlapping peptides included in the design. Bold residues indicate binding motif residues. The completeness score ranges between 0 and 1 and a higher score indicates more informative DMS data.

### 2.2 DMS by phage display correctly defines known SLiM consensuses

The DMS‐BM library was used in phage selections (Figure 1d) against the 11 different bait domains (Table 1; Table S2) resulting in binding enriched phage pools. The peptide‐coding regions of enriched phage pools from day 1 to day 4 of selections were barcoded and analyzed by NGS. The resulting DNA sequences were translated into peptide sequences, associated with their respective read counts (Table S3) and position specific scoring matrices (PSSMs) were generated for each bait‐peptide pair and each selection day. The results were evaluated to choose the appropriate round(s) of selection on which to generate the PSSMs. To this end, we used a benchmarking set of 234 PSSMs based on consensus aligned peptides from motif classes available in the ELM database (Kumar et al., 2024; Tsitsa et al., 2024). The information gathered in ELM is based on the manual curation of peptides binding to the domains from the literature and thus provides a solid publicly available reference set, with the limitation that the number of curated instances of binding peptides for each domain is limited and that the sequences of the potentially biologically relevant binders listed may not correspond to the biophysically best ligands. Indeed, endogenous ligands may have evolved to be suboptimal binders for functional reasons, while phage display often enriches for more hydrophobic peptides (Luck & Trave, 2011). Thus, some discrepancies between the PSSMs generated based on the information available in ELM and the PSSMs generated by the DMS analysis of a specific peptide sequence can be expected. The similarities between the ELM‐based PSSMs and the PSSMs defined by the DMS‐BM selection results were assessed for each phage selection round (i.e., after 1, 2, 3 and 4 days of selection), as well as for combined selection round results (that is for round 1–2, 2–3, 3–4, 1–3, 2–4, and 1–4). The effect of normalizing the results by library input was also assessed, as some correlation was observed between the peptide abundances in the input library and the outcome of the results of the first day of selection for some of the selections (Table [Link], [Link]). For each DMS‐based PSSM, a similarity score *p*‐value and the similarity rank of the PSSM for its corresponding ELM‐based PSSM in comparison to the remaining ELM classes screened were calculated (Table S5). To identify the optimal experimental setup, we benchmarked the various selections using a receiver operating characteristic (ROC) analysis of the PSSM similarity score *p*‐value and the rank of the true positives ELM classes (True Positives) in relation to the remaining ELM classes (False Positives). The area under the curve (AUC) was calculated (Figure 1e). We found that the quality of the PSSMs varied by the selection day and that the normalization step slightly improved the results for selection day 1 (Figure S1). However, the most informative results were obtained by combining the data of the second and third rounds of selections using the non‐normalized data (AUC 0.91 for the rank and 0.9 for the PSSM similarity), and we thus used these data for the further analysis (Figure S2 and Table S6). Notably, the results of the selection days 2 and 3 were almost as informative by themselves based on the AUC (Day 2: AUC 0.91 for the rank and 0.89 for the PSSM similarity; Day 3: AUC 0.91 for the rank and 0.88 for the PSSM similarity), and the DMS analysis may thus be conducted using only the results of the second or third day of selection. For each PSSM, a completeness score was further calculated and was found to be relatively high (Table 1). A high completeness score (close to 1) indicates that sequencing data generated from the binding‐enriched phage pools contained a high proportion of the designed mutations.

**FIGURE 2:**
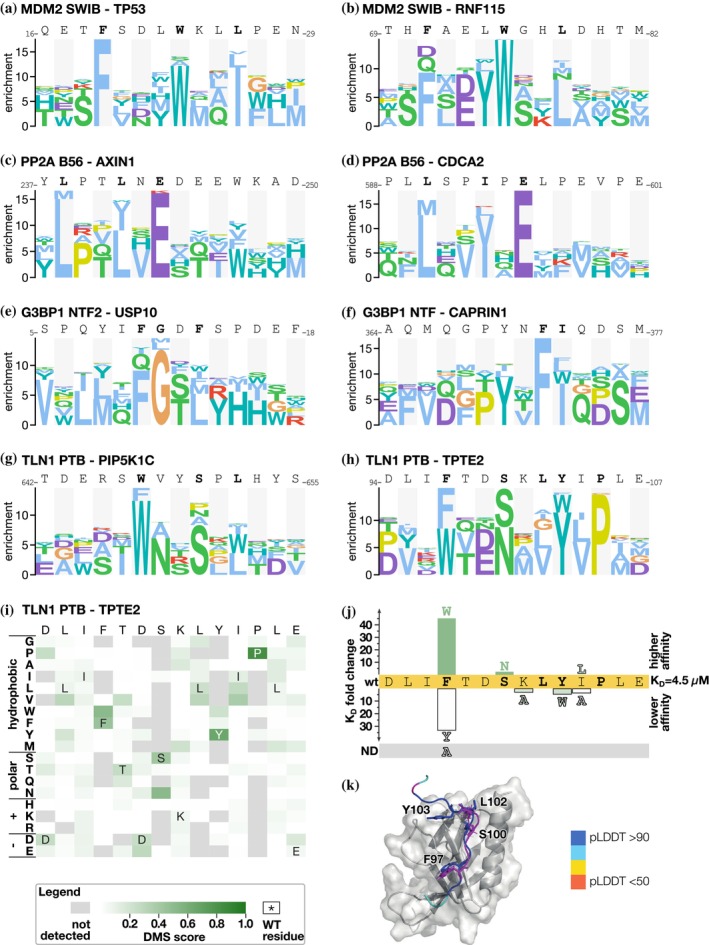
Examples of PSSMs generated by selections against the DMS‐BM library together with validation of an extended TLN1 PTB binding motif in TPTE2. (a‐h) Representative examples of PSSMs generated for MDM2 (a, b), PP2A B56 (c, d), G3BP1 (e, f), and TLN1 (g, h). (i) Heatmap representation of the PSSMs generated for the TPTE2 peptide binding to TLN1 PTB. The “DMS score” indicates the residue frequency from the PSSM. (j) Fold‐change of affinities of TLN1 PTB‐binding TPTE2 peptides upon mutation as determined using fluorescence polarization‐based affinity measurements. (k) AlpaFold3 model of the TLN1 PTB–TPTE2 complex overlayed with the previously solved NMR structure of TLN1 PTB in complex with PIP5K1C (PDB ID 2G35; peptide in magenta). The TPTE2 peptide is colored according to the pLDDT score, which shows the high confidence of the model (dark blue very high confidence pLDDT >90; light blue pLDDT 90 > pLDDT >70).

We compared the PSSMs generated based on the selection results for a given bait‐peptide pair against PSSMs for (i) the same bait with the overlapping peptide; (ii) PSSMs for the same bait with a distinct peptide; and (iii) PSSMs for bait–peptide pairs from unrelated baits. As expected, we observed the highest PSSM similarity for overlapping peptides, followed by distinct peptides binding the same bait, and finally, limited similarity with other PSSMs in the dataset (Figure 1f). The DMS‐based PSSMs generally encode binding determinants that are similar to the binding determinants described in ELM. The ELM‐derived PSSMs for the respective baits are the best matches for the DMS‐derived PSSMs for about 55% of the cases, including KEAP1 Kelch TGE motif and the EIF4E ligand (Table S5). Other representative examples include the DMS of the p53 and RNF115 peptides binding to MDM2, which resulted in the expected FxxxWxxL motif (Figure 2a,b). Similarly, the DMS of the PP2A B56 binding peptides from AXIN1 and CDC2A resulted in a [LM]xx[ILV]xE motif, which closely resembles the previously reported B56‐binding LxxIxE motif (Figure 2c,d) (Hertz et al., 2016; Wu et al., 2017). In other cases, we find that the ELM‐derived PSSMs are not representative of the motifs found in the peptides analyzed. For example, we note the PSSMs of the talin (TLN1) phosphotyrosine‐binding (PTB) domain for which the ELM class LIG_PTB_Apo_2 domain is a poor match with the observed motif as TLN1 PTB does not bind classical PTB‐binding motifs, as detailed later. For some baits, such as the G3BP1 NTF2‐like domain, we noted differences between the PSSMs generated using distinct model peptides. We used two distinct model peptides for G3BP1, one from USP10 and one from CAPRIN1, that are known binders of the same pocket but have distinct binding modes as shown by co‐crystallization of the complexes (Schulte et al., 2023; Song et al., 2022). The DMS analysis of the G3BP1 binding USP10 peptide (_3−_ *LH*SPQYIFGDFSPDEF*NQ*_−20_) correctly identified its G3BP1 binding FG motif (Figure 2e). The DMS analysis of the CAPRIN1 peptide (_362−_ *LM*AQMQGPYNFIQDSM*LD*_−379_) resulted in a distinct YxFI motif based on the averaged results of the two overlapping parental peptides. Notably, for the CAPRIN1_364‐379_ peptide which generated the highest quality data (completeness score 0.87), an extended YxFxxxSxL motif was obtained (Figure S2). This is similar to the extended YNFIxxxxL G3BP binding motif previously observed based on structural analysis (Schulte et al., 2023). As the terminal leucine is missing in the first CAPRIN1_362‐377_ peptide, the resulting averaged motif is truncated, suggesting that the frame of the peptides used may affect the motif observed. The results demonstrate the potential of the DMS by phage display approach to reveal distinct motifs within different bound conformations of peptide backbones, and to uncover ELM classes that might benefit from further curation.

### 2.3 DMS refines the talin‐1 PTB‐binding determinants

For the atypical PTB domain of TLN1, we probed the two distinct peptides, one from the phosphatidylinositol‐4‐phosphate 5‐kinase type‐1 gamma (PIP5K1C: _640−_FPTDERSWVYSPLHYS_−657_) and one from the transmembrane phosphoinositide 3‐phosphatase and tensin homolog 2 (TPTE2: _92−_LADLIFTDSKLYIPLEYR_−109_). Neither of these peptide sequences matches the ELM consensus for unphosphorylated PTB domain ligands (x[ILVMFY]xN.x[FY]x). The DMS analysis revealed a common consensus motif found in the two peptides, [WF]xxSxL, which in the TPTE2 peptide takes an extended form of FxxSxLYxP (Figure 2g,h). The TPTE2 peptide has a phenylalanine instead of a tryptophan at the first position of the motif, as compared to the higher affinity PIP5K1C ligand. We therefore determined the affinities for the wild‐type TPTE2_92‐107_ peptide, and its F97W, F97Y, and F97A mutants using a fluorescence polarization (FP)‐based assay (Figures S3 and S4; Table S7). While the wild‐type TPTE2_92‐107_ peptide is bound with a *K*_D_ value of 4.5 μM, the F97W mutant is bound with 44‐fold higher affinity (*K*_D_ = 0.1 μM; Figure 2j). The F97Y mutation conferred instead a reduced affinity (*K*_D_ = 150 μM; 33‐fold loss) while the F97A mutation resulted in loss of binding within the affinity range tested, highlighting the importance of the position for binding. The DMS analysis of the TPTE2 peptide further suggested that an asparagine would be well tolerated at the third position of the motif, and we found that a TPTE2_92‐107_ S100N mutation conferred a minor increase in affinity (S100N; *K*_D_ = 1.7 μM). We further explored the relevance of the putative extended motif in the TPTE2 peptide and found that mutation of Y103W (*K*_D_ = 22 μM) and I104A (*K*_D_ = 18 μM) reduced the affinity about fourfold, while a conservative I104L mutation had minor effects, supporting that the TPTE2 exploits a longer motif, that is, that the motif‐flanking region in the TPTE2 peptide contributes to binding. To gain further insight into how the TPTE2 peptide is bound by the TLN1 PTB domain, we modeled the complex using AlphaFold3 (Abramson et al., 2024) and overlaid it with the solved structure of the TLN1 PTB–PIP5K1C complex (Figure 2k; Figure S5). The structural analysis showed that the [WF]xxSxL part of the two peptides binds in a similar way, with the [WF] at the first position docking into a hydrophobic pocket. While the PIP5K1C peptide loops out from the binding site, the C‐terminal residues of TPTE2 peptide make additional contacts with the domain. In particular, Y103 fits into a shallow pocket at the lid region. Taken together, based on the DMS analysis we define a TLN1 PTB domain consensus motif, that can be C‐terminally extended.

### 2.4 Exploring SLiM‐based host‐virus interactions by DMS by phage display

**FIGURE 3:**
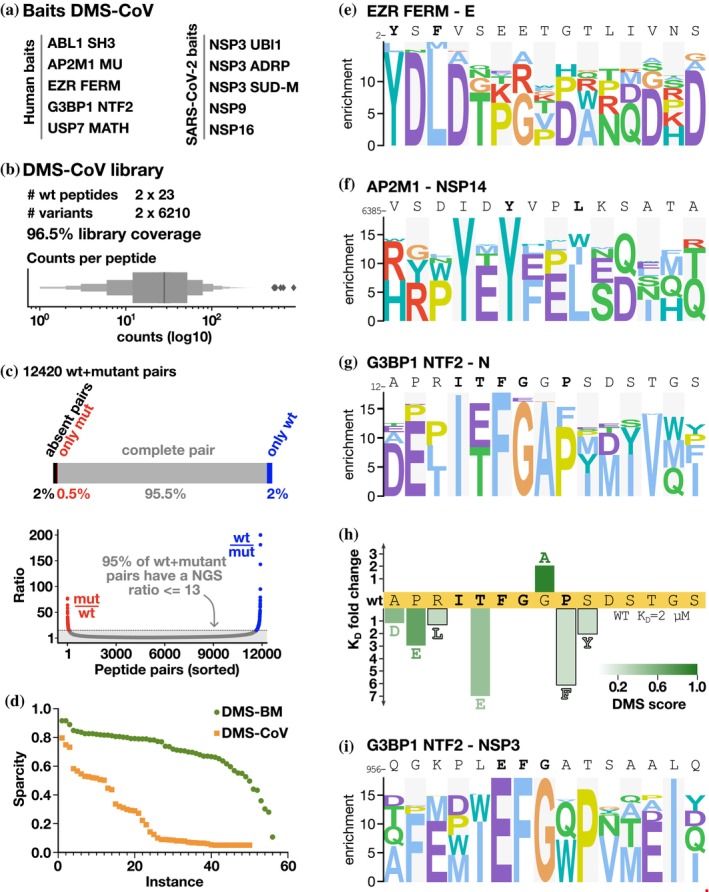
DMS‐CoV library quality and results for selections against human bait proteins. (a) Bait proteins selected for the DMS‐CoV analysis. (b) DMS‐CoV phage library design parameters, coverage, and counts distribution of all unique peptides. (c) DMS‐CoV library coverage on the wt/mutant peptides pair level. For 4% of the wt‐mutant peptide pairs, the mutant peptides were not found, and for 2.5% of the pairs, the wt peptides were also lacking, resulting in 2% of the pairs being absent from the physical phage library (top). The read count ratios for each wt/mutant peptide pair were evenly distributed, with the majority of the pairs having ratios of 13–1 or lower (bottom). (d) Completeness of DMS‐CoV selection results as compared to the DMS‐BM results. A low completeness score (*y*‐axis) indicates that sequencing data is missing for many mutations and amino acid positions for a given parent peptide (instance, *x*‐axis). (e–g, i) Representative PSSMs generated for viral peptides binding to the human bait protein domains EZR FERM (e), AP2 M1 (f), and G3BP1 NTF2 (g, i). (h) Fold‐change of affinities of G3BP1 NTF2‐binding N peptides upon mutation.

Having benchmarked the DMS by phage display protocol and showed its potential for uncovering novel details of well‐studied interactions, we next applied the approach to less explored host‐pathogen interactions. We designed a second DMS library (Table S1), termed DMS‐CoV, based on eight viral peptides binding to five human bait protein domains and eleven human peptides binding to five SARS‐CoV‐2 bait protein domains (Table 2). The studied interactions included, among others: two viral peptides binding to the G3BP1 NTF2‐like domain and human ligands of the globular domains of NSP3 and NSP9 (Figure 3a). The interactions were previously found through proteomic peptide phage display (Kruse et al., 2021; Mihalic Benz, et al., 2023) or predicted as binders based on the presence of a consensus binding motif (i.e. the SH3 binding PxxP motif in the SARS‐CoV‐2 N binding to ABL2 SH3 domain). NGS analysis confirmed that 96.5% of the designed oligonucleotides were represented in the constructed library (Figure 3b). While the sequence coverage was high, there were systematic deviations such that the SH3 binding _359−_DAYKTFPPTEPKKDKKKK_−376_ peptide from the N protein and its variants were depleted in the constructed phage library, possibly due to the lysine‐rich peptide interfering with phage virion assembly. Thus, the coverage of the DMS‐CoV library at the peptide level was lower than that of the DMS‐BM library (Figure 3c). Nevertheless, we used the DMS‐CoV library in selections against the defined bait collection. The results obtained using the DMS‐CoV library were less informative than the results obtained using the DMS‐BM library (Figure 3d), partially due to the lower coverage but likely also due to the fact that several of the interactions probed were of fairly low affinity, and in particular those between viral protein domains and human peptides (Mihalic, Benz, et al., 2023) and due to some specific traits of the motifs. In several cases, only one of the two overlapping parental peptides returned sufficient data, which may indicate that parts of the motifs were truncated in the shifted peptides. For example, for the EZR FERM domain, the analysis correctly identified the YxΦ motif in the N‐terminal part of the envelope (E) protein peptide (Figure 3e). The motif is lost in the shifted peptide, which explains the lack of information obtained for the second parental peptide tiling the region. Finally, the viral USP7 MATH domain ligands included in the design failed to be enriched in the selections as they were outcompeted by peptides from the MBOAT1 and AZIN2, which turned out to contain uncharacterized USP7 binding motifs (Figure S6). The results highlight that factors such as the affinity of the interactions probed and the position of the motif in the peptide should be considered when designing libraries for multiplexed DMS by phage display experiments.

**TABLE 2:** Overview of domains and peptides used for the design of the DMS‐CoV library, together with the indication of the outcome of the analysis as evaluated by the completeness score.

Domain	Peptide gene name	Peptide sequences	Completeness of DMS results for peptide 1/peptide 2
ABL2 SH3_444–508_	N	_359−_ *DA*YKTF**PP**TE**P**KKDKK*KK*_−376_	0.05/−
AP2M1 MU_160–435_	NSP14	_6384*−*_ *QV*VSDID**Y**VP**L**KSATA*IT*_−6401_	0.55/0.58
EZR FERM_2–295_	E	_1−_ *M**Y*** S**F**VSEETGTLIVNS*VL*_−18_	0.79/0.07
G3BP1 NFT2_1–139_	N	_11−_ *RN*APR**I**T**FG**GPSDSTG*SN*_−28_	0.13/0.60
	NSP3	_956−_ *YQ*GKP**L**E**FG**ATSAALQ*PE*_−973_	0.24/0.08
USP7 MATH_53–206_	N	_404−_ *DF*SKQLQQSMSSADST*QA*_−421_	0.05/0.05
	NSP3a	_238−_ ***P**V* **E**T**S**NSFDVLKSEDA*QG*_−255_	−/0.05
	NSP4	_3248−_ *VL*YQP**P**QT**S**ITSAVL*QS*_−3265_	−/0.05
NSP3 UBl1_819–925_	NCOA2	_1072−_ *PS*DEGAL**L**DQL**Y**LALR*NF*_−1089_	0.31/0.29
	NYNRIN	_1031−_ *EA*PSLSEEILRALSLH*DP*_−1048_	0.08/0.10
NSP3 ADRP_1023–1192_	AZIN2	_1−_ *MA*GYLSESDFVMVEEG*FS*_−18_	0.56/0.09
	MBOAT1	_16−_ *TG*STYL**HPLSE**LLGIP*LD*_−33_	0.34/0.55
NSP3 SUD‐M_1356–1493_	PRDM14	_197−_ *QF*TEEDLHFVLYGVTP*SL*_−214_	0.35/0.07
	TET3	_459−_ *DP*MAE**LE**Q**LLG**S**A**SD ***YI**Q*_−476_	0.06/0.07
NSP9_4141–4253_	AXIN1	_1−_ *MN*IQEQGF**GF**P**LD**LGASF*TE*_−18_	0.61/0.47
	NEK9	_737−_ *NS*S**GL**S**IG**TVFQSSSP*GG*_−754_	0.46/0.15
	NOTCH4	_1604−_ *TF*Q**GA**W**LG**APEPWEPL*LD*_−1621_	0.20/0.77
NSP16_6799–7088_	DYRK1B	_394−_ *EP*GHSPADYLR**F**QDLV*LR*_−411_	0.09/0.31
	ICA1L	_452−_ *QD*MSA**W**FNL**F**ADLDPL*SN*_−469_	0.53/0.09

*Note*: Italic indicates residues that are found only in one of the two overlapping peptides included in the design. Bold residues indicate binding motif residues based on consensus motifs or previous alanine scanning SPOT array analysis. The completeness score ranges between 0 and 1 and a higher score indicates more informative DMS data.

### 2.5 DMS results for SARS‐CoV‐2 peptides binding to the NTF‐like domains of G3BP1

Among the viral peptides binding to human proteins, we found two interactions particularly interesting. Firstly, our analysis confirmed the expected YxxL AP2M1 binding motif in the probed peptide from NSP14 (Figure 3f), but also suggested that an additional AP2M1 motif can emerge in the peptide upon an isoleucine to tyrosine substitution (YxxV), resulting in two potentially overlapping AP1M1 binding sites in the same peptide. Secondly, the DMS analysis correctly showed that the two viral G3BP1 binding peptides, N_11–28_ (*K*_D_ = 2.3 μM) and NSP3_956–973_ (61 μM) (Kruse et al., 2021) share an (E/T)FG motif (Figure 3g,i), similar to the FG motif found in USP10 (Figure 2). A T16E mutation in the N peptide conferred a sevenfold loss of affinity, partly explaining the higher affinity of the N peptide for G3BP1 as compared to the NSP3 peptide (N_12–26_ T16E = 16 μM; Figure 3h). In addition, the DMS results for the N peptide suggested that the interaction is supported by motif flanking residues (ITFGxP), which is consistent with the binding determinants (ITFG) resolved through co‐crystallization of the G3BP1 NTF‐N peptide complex (Biswal et al., 2022). The results further indicated that the proline contributes to binding, as a P20F mutation conferred a sixfold loss of affinity for N_12‐26_. The DMS results further suggested that the affinity of the N peptide for G3BP1 could be improved by mutating a glycine in a wild‐card position to alanine, and affinity measurements confirmed that the G19A mutation conferred a twofold increase in affinity (*K*_D_ wildtype N_12–26_ = 2.3 μM; N_12–26_ G19A = 1.1 μM). Other mutations tested in the flanking residues conferred moderate or minor losses of affinity (Figure 3h).

### 2.6 Refining the motifs in human peptides binding to protein domains from SARS‐CoV2 proteins

We next turn to the analysis of human peptides binding to viral protein domains expressed by the SARS‐CoV‐2 genome. We previously uncovered peptide‐based interactions of three NSP3 domains, NSP9, and NSP16 (Mihalic, Benz, et al., 2023), which were further explored here.

#### 2.6.1 NSP3

**FIGURE 4:**
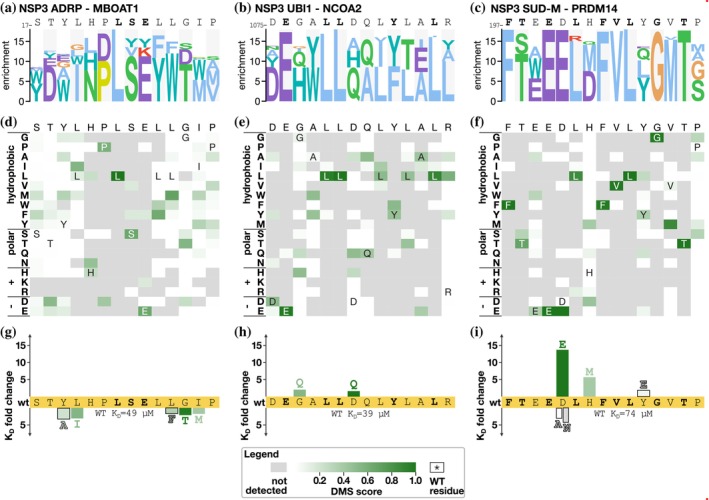
DMS analysis of peptides binding to the ADRP, UBl1, and SUD‐M domains of NSP3 PSSM (a–c) and heatmap (d–f) representations of the DMS data for the MBOAT1 peptide binding to NSP3 ADRP (a, d), the NCOA2 peptide binding to NSP3 UBl1 (b, e), and the PRDM14 peptide binding to NSP3 SUD‐M (c, f). (g–i) Fold‐change of affinities upon mutation of the respective wild‐type peptide binding to NSP3 ADRP (g), UBl1 (h), and SUD‐M (i).

The large multidomain protein NSP3 has several peptide‐binding domains including NSP3 ADRP, NSP3 UBl1, and NSP3 SUD‐M. For NSP3 ADRP, we tested two peptides previously identified as binders (AZIN2_1–18_ and MBOAT1_16–33_). Our previous alanine scanning SPOT array analysis suggested the core motif in the MBOAT peptide to be HPLSE (Mihalic, Benz, et al., 2023), and the current DMS analysis confirmed that this is a critical region for binding (Figure 4a,d). Based on the DMS results, we attempted to improve the affinity of the MBOAT1 peptide for NSP3 ADRP by a set of point mutations in the flanking regions, but the mutations resulted in minor (2–3 fold) losses of affinity in comparison to the wild‐type MBOAT1_16–31_ peptide (*K*_D_ = 49 μM) (Figure 4g, Figure S4, and Table S7).

For the NSP3 UBl1 domain, we also subjected two peptides to DMS analysis (NYNRIN_1031–1048_ and NCOA2_1072–1089_), of which NCOA2 is the higher affinity ligand (Mihalic, Benz, et al., 2023). Consistent with its higher affinity, the most informative results were obtained for the NCOA2_1072–1089_ peptide (Figure 4b,e) that converged on an extended ExxLLxxxYxxL motif. The extended motif partially matches the LxxxY motif previously suggested based on SPOT array alanine scanning (Mihalic, Benz, et al., 2023). In an attempt to increase the affinity of the interaction, we tested two mutations (G1077Q and D1081Q) and evaluated their effects on binding. Each of the mutations conferred minor increases in affinity in comparison to the wild‐type peptide (Figure 4h; *K*_D_ = 19 and 24 μM for G1077Q and D1081Q, respectively, in comparison to 39 μM for wild‐type NCOA2_1073–1088_).

For NSP3 SUD‐M, we tested the two model peptides, PRDM14_197–214_ and TET3_459–476_, of which the PRMD14_197–214_ peptide (_197−_ *QF*TEEDLHFVLYGVTP*SL*_−214_) is the higher affinity ligand (Mihalic, Benz, et al., 2023). Consistently, the DMS selection was dominated by the PRDM14 peptide and its variants (Figure 4c,f). The DMS analysis suggested that the peptide contains an extended FxxExLxFVLxGxT motif, which is similar to the previous results obtained through SPOT array alanine scanning (underlined; Mihalic, Benz, et al., 2023). We designed two mutations to improve the affinity of the interaction (D201E, H203M) and also tested a Y207E thought to be largely neutral to binding, and, as a control, included mutations that were expected to decrease the affinity (D201A, D201N). Affinity measurements revealed that the conservative D201E mutation had the most beneficial impact on binding (*K*_D_ = 5.4 μM, 14‐fold increase in affinity compared to wild‐type peptide *K*_D_ of 74 μM), followed by the H203M (*K*_D_ = 13 μM). The Y207E mutation also led to a minor improvement of affinity (*K*_D_ = 40 μM) (Figure 4i, Figure S4, and Table S7). As expected, the D201A and D201N mutations conferred reduced affinity.

In summary, the DMS analysis of the peptides binding to the NSP3 domains validated their key residues and pinpointed ways to improve their affinities, in particular, for the NSP3 SUD‐M domain.

#### 2.6.2 NSP9

**FIGURE 5:**
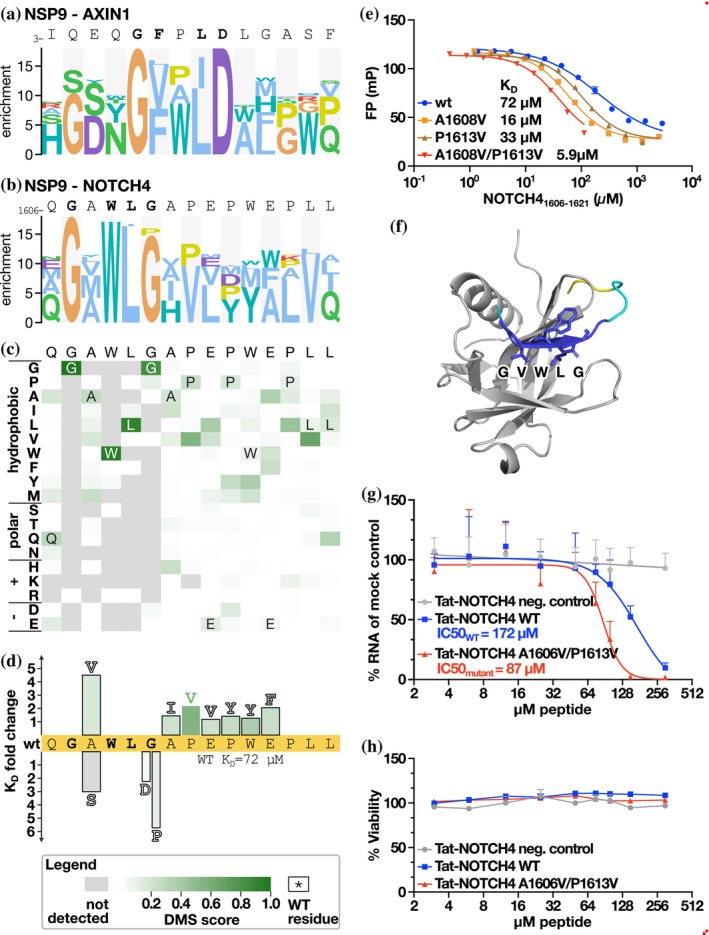
DMS analysis of NSP9‐binding peptides guides the engineering of more potent antiviral inhibitors. (a) PSSM representation of the DMS results of the Nsp9 binding AXIN1 peptide. (b, c) PSSM and heat map representation of the DMS results of the NSP9 binding NOTCH4 peptide. (d) Fold‐change of affinities upon point mutation of the NOTCH4 peptide. (e) FP‐based affinity determinations of wt, single (A1603V and P1613V) and double mutants of the NOTCH4 peptide binding to NSP9 (A1603V/P1613V). (f) AlphaFold3 model of the complex of NSP9 and the NOTCH4 A1603/P1613V peptide. Peptide coloring is according to the pLDDT score (deep blue = high confidence). (g) Evaluation of the antiviral effect of cell‐permeable Tat‐tagged variants of the NOTCH4 peptides in VeroE6 cells. VeroE6 cells were infected with SARS‐CoV‐2 (multiplicity of infection: 0.5) and 8 h post‐infection viral RNA was quantified using qPCR. Viral RNA was normalized to the RNA levels in mock‐treated cells and presented as % RNA of control. Data are cumulative of two independent experiments done in triplicates (*N* = 6). (h) Unaffected cell viability upon treatment with the Tat‐tagged peptides. Cellular viability after peptide treatment was measured using Celltiter Glo. Data are cumulative of two independent experiments done in triplicates (*N* = 6).

NSP9 is a component of the SARS‐CoV‐2 5′ mRNA capping machinery. We previously reported that it binds to a large number of peptides from human proteins containing a GΦxΦ[GD] motif, where Φ is a hydrophobic amino acid (Mihalic, Benz, et al., 2023), and these interactions were recently confirmed by surface plasmon resonance experiments (Baker et al., 2024). Here, we probed its binding to three peptides, from AXIN1, NEK9 and NOTCH4, respectively, where analysis of AXIN1 and NOTCH4 peptides produced the most robust data. The DMS analysis of the AXIN1 peptide resulted in a G[VF]x[IL]D motif (Figure 5a), while the NOTCH4 peptide instead revealed a similar yet distinct GxWLG motif (Figure 5b,c). Affinity measurements of the wild‐type and mutant NOTCH4 peptides and NSP9 showed that a glycine to aspartic acid (G1611D; *K*_D_ = 160 μM), or a glycine to proline (G1611P; *K*_D_ = 410 μM) substitution at the last position of the motif conferred losses of affinity (2‐ to 5‐fold) as compared to the affinity for the wild‐type NOTCH4 peptide (*K*_D_ = 72 μM), thus supporting the motif variations between the two model peptides (Figure S4 and Table S7).

As the NSP9‐binding NOTCH4 peptide has previously been shown to have an antiviral effect (Mihalic Benz, et al., 2023), we further attempted to generate a higher affinity ligand based on the DMS data. We tested seven mutations and found each of them to confer minor increases in affinities, with a conservative A1608V mutation at the second position of the motif having the largest effect (A1608V, *K*_D_ = 16 μM versus *K*_D_ = 72 μM for the wild‐type; Figure 5d,e). As a negative control, we tested an A1608S mutation, which as expected led to a decreased affinity (*K*_D_ = 220 μM). We combined the A1608V mutation with a P1613V mutation (*K*_D_ = 33 μM) into a double mutant, which resulted in a further increase in affinity (A1608V/P1613V *K*_D_ = 5.9 μM). Having generated a higher affinity NSP9 ligand, we used AlphaFold3 (Abramson et al., 2024) to model the complex. In contrast to the wild‐type peptide, the NOTCH4 A1608V/P1613V variant was confidently docked, with the model suggesting that the GVWLG part of the peptide binds through beta‐strand addition (Figure 5f). The proposed NSP9 binding region coincides with residues previously mapped by NMR to be perturbed by peptide binding (Mihalic, Benz, et al., 2023). Thus, the previously NMR‐mapped binding site and the AlphaFold3‐based model confidently pinpoint the peptide binding region on NSP9.

Finally, we tested the antiviral activity of the affinity‐matured NOTCH4 A1608V/P1613V peptide by fusing it to a cell‐penetrating Tat‐tag and evaluated its antiviral effect in comparison to the Tat‐tagged NOTCH4 wild‐type peptide. VeroE6 cells were treated with the Tat‐tagged peptides and infected with SARS‐CoV‐2 (multiplicity of infection: 0.5). The viral RNA was quantified 8 h post‐infection using qPCR. The analysis showed that the Tat‐tagged NOTCH4 A1608V/P1613V peptide is a more potent antiviral inhibitor than the wild‐type peptide, consistent with the higher affinity (Figure 5g), while not having any effect on cell viability (Figure 5h).

## 3 DISCUSSION

In this study, we outline a multiplexed DMS by peptide‐phage display protocol. Through benchmarking the results against a set of previously defined motifs reported in ELM (Kumar et al., 2024), we show that DMS by phage display is an efficient approach for defining an interaction motif consensus. The phage display‐derived DMS data can identify the key residues in the motif and define preferred amino acids in these positions in the context of the chosen peptide. In addition to consensus discovery, we find that the DMS analysis pinpoints variations of the motifs that are not captured by the general motif descriptions or from consensus motifs generated by aligning cohorts of binding peptides. For example, we highlight the case of the TLN1 PTB domain, for which we defined a general consensus motif ([WF]xx[SN]x[ILV]), which can be supported by a C‐terminal extension ([WF]xx[SN]x[ILV]xYxP). The two similar yet distinct motifs found in the G3BP1‐binding peptides from USP10 and CAPRIN1 also support this point.

We further applied the “DMS by phage display” protocol on less explored SLiM‐based host‐virus interactions. This analysis turned out to be more challenging, likely due to the lower affinities of the interactions. Nevertheless, the DMS analysis confirmed and substantiated the previously described motifs binding to the SARS‐CoV‐2 domains NSP3 UBl1, NSP3 ADRP, NSP9, and NSP16. The data also revealed detailed motif variations in the peptides binding to NSP9. The results support that the general NSP9 binding motif is GΦxΦ[GD] and that the NOTCH4 peptide has a similar yet distinct motif (GxWLG). The two variant motifs dock to the same site based on AlphaFold3 modeling (Figure S7), and the motif variation appears to be caused by the requirements posed by the need to accommodate a bulky tryptophan in the NOTCH4 upon beta‐strand addition. Moreover, we showcase how DMS by phage display can be used to increase the affinity of peptide ligands, and that the affinity‐matured NSP9 binding peptide has increased antiviral activity. Consequently, DMS by phage display can be used both to identify the determinants of host‐virus protein–protein interactions and to increase the affinity of peptide ligands as a part of peptide‐based inhibitor development.

A strength of DMS by phage display is the scalability as it can easily be performed in parallel for multiple peptides binding to various protein domains. Given the multiplexing possibilities, we envision the integration of DMS into a workflow where a limited set of ligands has been identified for several different SLiM‐binding protein domains. We note that there are many different ways of exploring the results. Based on the benchmarking, we chose a general approach where we combine the outcome of the second and third selection days, which we find provide a robust generally applicable protocol. However, the outcomes of the second and third days of selections were almost of equal quality as the pooled results of the two days, and it would also have been valid to base the analysis on the results of either the second or the third day of selections. We further note that deep mutational scanning protocols commonly apply input library normalization on the output. However, our benchmarking suggested that such normalization did not improve the results except for the results generated for the first day of selection. This can be explained by the fact that our input library was fairly balanced (Figure 1c) and that the input bias is not propagated to the outcome of the later selection days due to the strong selection pressure for binding. However, if using the outcome of the first selection day only, then it would have been appropriate with normalization for the naïve library input bias.

The DMS by phage display builds on the assumption that there is a relation between the peptide enrichment revealed by NGS and the relative affinities. Although the data is somewhat noisy, we find by plotting the NGS counts versus the affinities that this is to a certain extent a valid assumption for five out of six domain‐peptide systems tested (the exception being the NSP3 ADRP‐MBOAT1 interaction) (Figure S8). While it could be envisioned to correlate the observed NGS counts to affinities using a calibration curve of measured affinities, it does not appear feasible, given that the results are noisy and system dependent. Another limitation of the approach is that it does not perform well for low‐affinity interactions (e.g., with *K*_D_ values above 100 μM) and that care needs to be taken when designing the library (e.g., choice of model peptides). When combining multiple DMS analyses into one experiment, there is also the risk of unexpected competition between different ligands targeting the same pocket. An alternative approach to analyze multiple interactions by DMS could be to parallelize library making and phage display screening to have individual libraries for each domain. However, we find that the advantage of making one pooled library for many baits in comparison to making unique libraries for each bait outweighs such disadvantages, given the scalability of the approach. Also, the discovery of unexpected interactions and mutations that shift the target specificity of the peptides from one domain type to another may by itself provide valuable information related to SLiM evolution. With these limitations in mind, we conclude that DMS by peptide‐phage display can be applied to obtain information on binding determinants for multiple proteins in parallel and pinpoint the divergent affinity determinants in distinct peptide backgrounds. DMS by peptide‐phage display thus represents a viable addition to the toolbox for exploration of SLiM‐based interactions.

## 4 MATERIALS AND METHODS

### 4.1 Library design

The DMS‐BM and DMS‐CoV phage libraries were designed based on previously reported ligands (Benz et al., 2022; Kruse et al., 2021; Kumar et al., 2024; Mihalic, Benz, et al., 2023). Each wild‐type peptide was tiled by two overlapping peptides shifted by 2 amino acids. All wt peptides were mutated at 15 positions, including all overlapping positions to all‐natural amino acids except cysteine. The peptides were reverse translated to oligonucleotides optimized for *E. coli* expression, and flanking regions for library creation were added (5′ CAGCCTCTTCATCTGGC and 3′ GGTGGAGGATCCGGAG).

### 4.2 Phage library constructions

The oligonucleotides (GenScript) were PCR amplified with Phusion PCR Master Mix (Fisher Scientific) using 90 s 98°C initial denaturation; 18 cycles of 15 s 98°C denaturation, 15 s 55–58°C annealing, and 15 s 72°C elongation; and 5 min 72°C final elongation. The PCR products were purified using the MinElute PCR Purification Kit (Qiagen). The PCR‐amplified oligonucleotides (0.6 μg) were 5′ phosphorylated with 20 units of T4 polynucleotide kinase (Fisher Scientific) at 37°C for 1 h in 1× TM buffer (10 mM MgCl_2_, 50 mM Tris–HCl, pH 7.5) supplemented with 5 mM dithiothreitol (DTT) and 1 mM adenosine triphosphate (ATP). Following 5 min of cooling on ice, the oligonucleotides were annealed to 10 μg of dU‐ssDNA phagemid (90°C for 3 min, 50°C for 3 min, and 20°C for 5 min) in TM buffer. DNA polymerization and ligation were initiated by adding 10 μL 10 mM ATP, 10 μL 10 mM dNTP, 15 μL 100 mM DTT, 30 Weiss units of T4 DNA ligase (Fisher Scientific), and 30 units of T7 DNA polymerase (Fisher Scientific), followed by incubation at 20°C for 16 h. The reaction was stopped by three freeze–thawing cycles. Remaining wild‐type dU‐ssDNA was digested by incubating with 5 μL FastDigest SmaI (Fisher Scientific, 37°C, 30 min). dsDNA was purified using the QIAquick PCR & Gel Cleanup Kit (Qiagen). The dsDNA phagemid library was electroporated into *E. coli* SS320 cells (Lucigen) pre‐infected with M13KO7 helper phages (ThermoFisher). Electroporated cells were rescued in 25 mL pre‐warmed super optimal broth (SOC) medium (0.5 w/v% yeast extract, 2 w/v% tryptone, 10 mM NaCl, 2.5 mM KCl, 10 mM MgCl_2_, 10 mM MgSO_4_, and 20 mM glucose, pH = 7.0) and incubated at 37°C for 30 min. The phage‐producing bacteria were grown overnight (±18 h) in 0.5 L 2YT medium (1 w/v% yeast extract, 1.6 w/v% tryptone, and 0.5 w/v% NaCl) at 37°C, and then harvested. Phage libraries were stored at −80°C in 10 v/v% glycerol.

### 4.3 Bait expression and purification

The pETM33 (EMBL), PH1003 (Sidhu Lab), pET42a(+) (EMD Biosciences), or pGEX‐4T1 (GenScript) vectors containing cDNA encoding bait proteins (Table S2) were used to express GST‐tagged baits. Overnight cultures (2YT, 37°C, 200 rpm, 18 h) of *E. coli* Bl21 DE3 gold cells (Agilent) transformed with the appropriate vector were used to inoculate 500 mL 2YT (supplemented with Kan (50 μg/mL) or Carb (100 μg/mL)), followed by incubation (37°C, 200 rpm). Protein production was induced at OD_600_ 0.6–0.8, with 1 mM isopropyl β‐d‐1‐thiogalactopyranoside (IPTG) for 18–20 h at 18°C, 200 rpm. The bacteria were pelleted (5000*g*, 5–7 min) and stored at −20°C. Bacteria were dissolved in lysis buffer (phosphate buffered saline (PBS, 37 mM NaCl, 2.7 mM KCl, 8 mM Na_2_HPO_4_, 2 mM KH_2_PO_4_), pH 7.4, 1% Triton X‐100, 10 μg/mL DNase I, 5 mM MgCl_2_, lysozyme (Thermo Scientific), cOmplete Mini, EDTA‐free, Protease Inhibitor tablet (Roche, 1 tablet/10 mL)), incubated at 4°C for 1 h and sonicated (2 s pulse, 2 s pause for 20 s). Cell debris was removed (16,000*g*, 4°C, 1 h). The supernatant was incubated with glutathione (GSH) Sepharose 4 Fast Flow beads (Cytiva; 1 mL resin/bacterial pellet) (4°C, agitation, 1 h), the resin was pelleted by centrifugation (500*g*), the supernatant was removed and the beads were washed with PBS. Proteins were eluted stepwise with 1 mL elution buffer (50 mM Tris–HCl, 10 mM reduced glutathione, pH 8.0). Protein purities and sizes were confirmed through SDS‐PAGE (BioRad Mini‐PROTEAN TGX Stain‐Free Precast gels, 200 V, 30 min). Purified bait proteins were flash frozen using liquid nitrogen in 16 v/v% glycerol and stored at −80°C until further use.

### 4.4 Phage selections

Ten micrograms of GST‐tagged bait proteins or GST (negative control) in 100 μL PBS were immobilized in Nunc MaxiSorp flat‐bottom 96‐well plates (ThermoFisher Scientific, Cat: 44‐2404‐21) for 18 h at 4°C. Wells were blocked with 200 μL 0.5% bovine serum albumin (BSA) in PBS for 1 h at 4°C under gentle agitation. GST‐coated wells were washed four times with 200 μL PT (PBS + 0.05 v/v% Tween 20) and (naïve) phage library (10^11^ phages, 100 μL in PBS) was added to each GST‐coated well. Following incubation (4°C, gentle agitation, 1 h), the phage library was transferred to blocked and washed bait‐protein‐coated wells. After 2 h of incubation at 4°C, unbound phages were removed by five 200 μL PT washes. Bound phages were eluted with 100 μL log‐phase *E. coli* OmniMAX cells (cultured in 2YT medium supplemented with 10 mg/mL tetracycline) for 30 min at 37°C under gentle agitation. 10^9^ M13KO7 helper phages (ThermoFisher) were added to each well and allowed to infect bacteria for 45 min at 37°C. The hyperinfected bacteria were transferred to 1 mL 2YT supplemented with 30 μg/mL Kan, 100 μg/mL Carb, and 0.3 mM IPTG and incubated overnight (37°C, 200 rpm, ±18 h). Bacteria were pelleted (2000*g*, 4°C, 10 min) and the phage supernatants were transferred to a fresh 96‐deep‐well plate, pH adjusted by adding 1/10 volume 10× PBS, and heat‐inactivated through incubation at 65°C for 10 min. The phage pools were used for the next day of selection.

### 4.5 Phage Pool ELISA

Proteins (10 μg) in PBS (100 μL/well) were coated in Nunc MaxiSorp flat‐bottom 96‐well plates for 18 h at 4°C under gentle agitation. Wells were blocked with 200 μL 0.5% BSA in PBS (4°C, 1 h). Phages (100 μL) were allowed to bind to the bait protein‐ or GST‐coated wells for 1 h at 4°C. Unbound phages were washed away with 4× 200 μL PT, and 100 μL HRP‐conjugated anti‐M13 bacteriophage antibody was added (Sino Biological Inc., Cat: 11973‐MM05T‐H, 1:5000 diluted in 0.5% BSA in PT). Following a 1 h incubation at 4°C, wells were washed four times with 200 μL PT and once with 200 μL PBS. 100 μL TMB substrate (Seracare, Cat: 5120‐0047) was used to detect the bound antibody, and the enzymatic reaction was stopped through the addition of 100 μL 0.6 M sulfuric acid (H_2_SO_4_). The absorbance at 450 nm was measured with a SpectraMax iD5 microplate reader (Molecular Devices).

### 4.6 Sample preparation for NGS and data analysis

Peptide‐coding regions were amplified and barcoded using Phusion PCR Master Mix (Fisher Scientific) for 22 cycles. PCR products (25 μL) were normalized using Mag‐Bind Total Pure NGS magnetic beads (Omega Bio‐Tek, Cat: M1378). Normalized PCR products (10 μL) were pooled and purified using gel purification. DNA was eluted with 30 μL TE buffer (10 mM Tris–HCl, 1 mM EDTA, pH 7.5). The amplicon pool was sent for NGS (Illumina MiSeq v3, 1×150bp read setup, 20% PhiX, performed by the NGS‐NGI SciLifeLab facility). The raw NGS data were demultiplexed, sequencing adapters were trimmed, and the sequences with an average quality of 20 or more were then translated to peptide sequences. A table was generated containing all peptide sequences and their total read counts. No minimum count filtering was enforced, but only those peptides that were expected for the bait used in the selection were kept for the analysis (Table S3). The NGS sequence processing was performed using custom Python scripts.

A frequency PSSM (PSSMfreq) was built for each wildtype and its corresponding mutant peptides. First, the raw counts for each peptide were converted to fractional counts by dividing the raw counts by the total sum of counts for all peptides in each selection replicate. A normalized library input fractional count was also calculated by dividing the fractional counts in selection results by the fractional count observed in the coverage evaluation of the library. Then the fractional counts across replicates, and when applicable across selection days, were averaged to create a single averaged fractional count per peptide. The data were used to build a PSSM that was finally turned to a PSSMfreq by calculating the “DMS score,” or per column frequency values, for all 19 amino acid variants (no cysteine) at each position following Equation (1), where *count* refers to the averaged fractional count:

(1) $${\text{PSSM}}_{{\text{freq}}_{\left(c,i\right)}}=\frac{{\text{count}}_{\left(c,i\right)}}{\sum_{i=1}^{19}{\text{count}}_{\left(c,i\right)}}.$$

Logos were built from the PSSMs by calculating the relative enrichment of each amino acid at each position with Equation (2):

(2) $${\text{Relative enrichment}}_{\left(c,i\right)}=\frac{{\text{PSSM}}_{{\text{freq}}_{\left(c,i\right)}}-\text{expected frequency}}{\text{expected �frequency}},�$$

where the expected frequency for any amino acids was 1/19 (as Cysteines are not present in the library). The enrichment matrices were plotted as logos using Python's library Logomaker (Tareen & Kinney, 2020).

### 4.7 ELM instance specificity determinant dataset

A dataset of PSSMs encoding motif class specificity determinants was created from the motif instances in the ELM database (Kumar et al., 2024). For each SLiM class, peptides were extracted and aligned using the ELM‐defined class consensus; alignments were converted to a PSSM using the PSSMSearch web application (Krystkowiak et al., 2018) with default parameters and the frequency PSSM scoring method, resulting in 234 PSSMs. Each bait‐peptide pair screened in the DMS‐BM analysis was annotated with a corresponding ELM class. The dataset is available online (DOI: 10.5281/zenodo.15297110).

### 4.8 Specificity determinant comparison

The similarities between the specificity determinants resulting from the DMS‐BM screens and the expected specificity determinants were quantified using PSSM–PSSM comparison. The comparison was performed with the CompariPSSM tool, which slides two PSSMs across each other and calculates the similarity of each comparison window (Tsitsa et al., 2024). The similarity of each corresponding column in the window was calculated using Pearson's correlation. The importance of the amino acid position was also calculated for each column using the Gini coefficient, a measure of statistical dispersion that calculates the inequality among values. The importance‐weighted similarity score (ISW) is then calculated using Equation (3), where *n* is the number of positions in the motif alignment, *A*_i_ is the position *i* in PSSM_A_, and *B*_i_ is the position *i* in the PSSM_B_:

(3) $$\begin{array}{l} \text{Importance Weighted Similarity Score}\\ \;=\sum_{i=1}^{n}\left(\text{Similarity}\;A_{i}B_{i}\times \text{Importance}\;A_{i}\times \text{Importance}\;B_{i}\right). \end{array}$$

The probability of the observed importance‐weighted similarity score between two columns was calculated using a randomization approach based on the comparison of random PSSM columns. A sample of 100,000 randomly selected column pairs between the two PSSM datasets was compared, and the distribution of importance‐weighted similarity scores was calculated. The likelihood of seeing the observed importance‐weighted similarity score by chance, *p* ^*ISW*^, was defined based on the distribution of the importance‐weighted similarity score of the randomly paired PSSM columns. The probability of the window, *p* ^*window*^, was calculated as the product of the pairwise column *p* ^*ISW*^ probabilities from the window. The *p* ^*window*^ score was normalized to correct for the number of comparisons performed for the window using uniform product distribution correction to define the *p* ^*window_corrected*^ probability. After all comparison windows were scored, the highest‐scoring pair of windows was returned as the aligned specificity determinants, and the *p* ^*window_corrected*^ was used as the similarity score between the PSSMs.

### 4.9 Completeness

Completeness measures the proportion of cells in the PSSM where there is no data. A completeness of 1 denotes that all the cells in a PSSM have information, and a completeness of 0 denotes that there is no information in any of the cells in a PSSM. A completeness ratio was calculated using Equation (4), where $n_{0}$ is the number of cells in the PSSM with 0 value and *N* is the total number of cells in the PSSM:

(4) $$\text{Completeness}=1-\frac{n_{0}}{N}.$$

### 4.10 Establishing the optimal combination of selection days

All normalized and non‐normalized PSSMs split by the day of the selections (1, 2, 3, and 4) and combined day of the selections (1/2, 2/3, 3/4, 1/2/3, and 2/3/4) were compared with the dataset of 234 ELM class specificity determinant PSSMs. A similarity score *p*‐value and the similarity score‐derived rank of each comparison were calculated. The comparisons with the bait‐peptide pair PSSM with the expected ELM class were classified as True Positive, and all the other comparisons were classified as False Positives. For both the *p*‐value and the rank data, an ROC analysis was performed, and the area under the curve (AUC) was calculated to measure the quality of the selections on different days and combinations of days. The metrics were calculated using the *roc_curve* and *auc* functions from the *sklearn.metrics* library in Python 3.9.7, respectively.

### 4.11 Replicate comparison

The specificity determinants derived from overlapping and distinct peptide replicates were compared for each peptide in the DMS‐BM bait–peptide pair set and a comparison *p‐value* was calculated from each comparison. The *p*‐values for the comparison of the bait–peptide pairs were grouped based on the following criteria: (i) “Same bait/Overlapping peptide” for the bait‐peptide pair with the same bait and overlapping peptides, (ii) “Same bait–Different peptide” for the bait–peptide pair with the same bait and non‐overlapping peptides, and “Other” for the remaining peptides. “Same Bait–Same Peptide” were excluded from the analysis. The groups were then plotted as boxplots using the *seaborn 0.11.1* library in Python 3.9.7. The difference in the means of the groups was compared with a pairwise Mann–Whitney test using the *stats.mannwhitneyu* function from the *scipy 1.7.0* library in Python 3.9.7.

### 4.12 Expression and purification of proteins for affinity measurements

His‐GST‐tagged human bait proteins were expressed in 4 L *E. coli* BL21 (DE3) cultures for FP‐based affinity measurements. Lysates were cleared by centrifugation (16,000 RCF, 4°C, 1 h) and the supernatants were mixed with Ni Sepharose High Performance resin (Cytiva) (1 mL beads/pellet) followed by incubation (4°C, agitation, 1 h). The beads were washed with washing buffer (20 mM NaPO_4_, 0.5 M NaCl, 30 mM imidazole, pH = 7.5). The His‐GST tags were cleaved by incubation for 16–18 h at 4°C in 200 μL 0.5 mg HRV 3C protease and 1 mL primary buffer (20 mM NaPO_4_, 0.5 M NaCl, pH = 7.5). Cleaved proteins were collected. The samples were dialysed to 50 mM potassium phosphate buffer (pH = 7.5) for 16–18 h at 4°C. Protein purity and quality were confirmed through SDS‐PAGE and thermal shift assay (Tycho NT.6, NanoTemper). His‐GST tagged domains of SARS‐CoV‐2 proteins were expressed and harvested as described above. After the centrifugation step, the lysate was mixed with Pierce glutathione agarose (ThermoFisher) and incubated at 4°C under agitation for 30 min. The gel was washed with wash buffer 2 (50 mM Tris, 300 mM NaCl, 2 mM DTT, pH 7.8) and the protein of interest was eluted with elution buffer (wash buffer 2 supplemented with 10 mM reduced GSH). The His‐GST tag was cleaved using HRV 3C protease (inhouse; 18 h at 4°C) and the cleaved tag was removed by reverse immobilized metal affinity chromatography. Purified proteins were subjected to size exclusion chromatography (HiLoad 16/600 Superdex 75 pg.; Cytiva) to remove any residual impurities, concentrated, flash frozen, and stored at −80°C until further use.

### 4.13 FP‐monitored affinity measurements

FP‐monitored affinity measurements were carried out in triplicates with a SpectraMax iD5 Multi‐Mode Microplate Reader (Molecular Devices) in Corning 96‐Well Half‐Area Plates [Black, Flat‐bottom, non‐binding surface (Corning, Cat: 3993]), with excitation at 485 nm, emission at 535 nm in a total volume of 50 μL. Peptides were obtained at >95% purity (GeneCust). FITC‐labeled peptides were dissolved in dimethyl sulfoxide (DMSO) and diluted 1:1000 in 50 mM KPO_4_ buffer (pH 7.5). Unlabeled peptides were dissolved in a 50 mM KPO_4_ buffer (pH 7.5). Peptide concentrations were determined spectroscopically (FITC‐labeled peptides: *λ* = 495 nm, unlabeled peptides: *λ* = 280 nm). For saturation experiments, bait proteins in 50 mM KPO_4_ pH 7.5 solution (for SARS‐CoV‐2 protein domains the assay buffer was supplemented with 0.05% Tween20 and 1 mM TCEP) were arrayed in serial dilution (Diluent: 50 mM KPO_4_, pH = 7.5; for SARS‐CoV‐2 proteins same adjustment of buffer was made as described above): 25 μL protein solution followed by addition of 25 μL peptide master mix (2 mM DTT and 10 nM labeled peptide in 50 mM KPO_4_ buffer, pH 7.5). For the displacement experiments, unlabeled peptides were arrayed in serial dilution: 25 μL unlabeled peptide solution followed by addition of 25 μL peptide master mix supplemented with the protein of interest at a concentration of 4× the *K*_D_ value. Data were analyzed using GraphPad Prism (GraphPad Software, San Diego, California USA). *K*_D_ values from the direct binding experiments were obtained by fitting against a quadratic equation (Equation 5) for determination of *K*_D_ values for the direct FP‐monitored binding experiments. “pept” indicates the fixed probe peptide concentrations, *X* indicates the protein concentration, the constant *A* is the signal amplitude divided by probe peptide concentration, and *B* is the plateau value.

(5) $$Y=A\times \frac{\text{pept}+X+K_{D}+\sqrt{\left(\text{pept}+X+K_{D}\right)-4\;\text{pept}*X}}{2}+B.$$

The results of the FP competition experiments were fitted to a sigmoidal dose–response (variable response; GraphPad Prism).

### 4.14 Infections experiment

VeroE6 cells were infected with SARS‐CoV‐2 (MOI:0.5) for 1 h at 37°C and 5% CO_2_, then the inoculum was removed and replaced with a medium containing the indicated concentration of peptide. Eight hours post infection, cells were lysed and RNA was isolated using NucleoSpin RNA Plus XS (Macherey Nagel) according to the manufacturer's instructions. cDNA was synthesized using the High‐capacity cDNA Reverse Transcription kit (Thermo Fisher). SARS‐CoV‐2 RNA was quantified using qPCRBIO probe mix Hi‐ROX (PCR Biosystems) and the following primers and probes: GTCATGTGTGGCGGTTCACT, CAACACTATTAGCATAAGCAGTTGT, and FAM‐CAGGTGGAACCTCATCAGGAGATGC‐BHQ. GAPDH was used as a reference gene, detected by the RT qPCR Primer Assay (NM_001195426, Qiagen) and the qPCRBIO SyGreen mix Hi‐ROX (PCR Biosystems). qPCR experiments were run on a StepOnePlus real‐time PCR system (Applied Biosystems).

### 4.15 Viability test

Cells were treated with the indicated peptides for 8 h, then cellular viability was determined using CellTiter‐Glo® Luminescent Cell Viability Assay (Promega) on a Varioskan LUX Multimode Microplate Reader (ThermoFisher Scientific).

## AUTHOR CONTRIBUTIONS

**Caroline Benz:** Conceptualization; investigation; writing – original draft. **Lars Maassen:** Investigation. **Leandro Simonetti:** Investigation; visualization; writing – review and editing. **Filip Mihalic:** Investigation; writing – review and editing. **Richard Lindqvist:** Investigation; writing – review and editing. **Ifigenia Tsitsa:** Investigation; writing – review and editing. **Aimiliani Konstantinou:** Investigation. **Per Jemth:** Supervision. **Anna K. Överby:** Writing – review and editing; supervision; funding acquisition. **Norman E. Davey:** Conceptualization; investigation; writing – review and editing; funding acquisition. **Ylva Ivarsson:** Conceptualization; investigation; funding acquisition; writing – original draft; writing – review and editing; supervision.

## CONFLICT OF INTEREST STATEMENT

The authors declare no conflicts of interest.

## Supporting information


**Data S1.** Supplementary figures.

Table S1.

Table S2.

Table S3.

Table S4.

Table S5.

Table S6.

Table S7.

## Data Availability

The data that supports the findings of this study are available in the supplementary material of this article.
