# Supplementary material for: Defining short linear motif binding determinants by phage display‐based deep mutational scanning
Source: Protein Sci. 2025 May 24;34(6):e70174. doi: 10.1002/pro.70174 (PMC12102759; doi:10.1002/pro.70174)
Supplement: Supplementary file 1 — Data S1. Supplementary figures. [file PRO-34-e70174-s002.pdf]

Supplemental information for "Defining short linear motif binding determinants by phage-based multiplexed deep mutational scanning" by Benz et al.

Figure S1. ROC analysis to benchmark the DMS by phage analysis PSSMs created from the by input library normalized DMS-BM selection data, compared to PSSMs generated from peptide instances curated in the ELM database.

Figure S2. Heat map and PSSM representation of DMS analysis results generated through selections against the DMS-BM library.

Figure S3. Binding titration curves as detected by FP of FITC-labeled probe peptides.

Figure S4. Affinity determinations through fluorescence polarization-based competition experiments.

Figure S5. AlphaFold3 model of the TLN1 PTB-TPTE2 complex. Larger version of Fig. 1K.

Figure S6. Heat map and PSSM representation of DMS analysis results generated through selections against the DMS-BM library.

Figure S7. AlphaFold3 model of the complex of NSP9 and the NOTCH4 A1603V/P1613V peptide (QGVWLGAVEPWEPL) overlayed with the model of the AXIN (IQEQGFGFPLDLGAS).

Figure S8. Correlation between NGS counts and affinities.

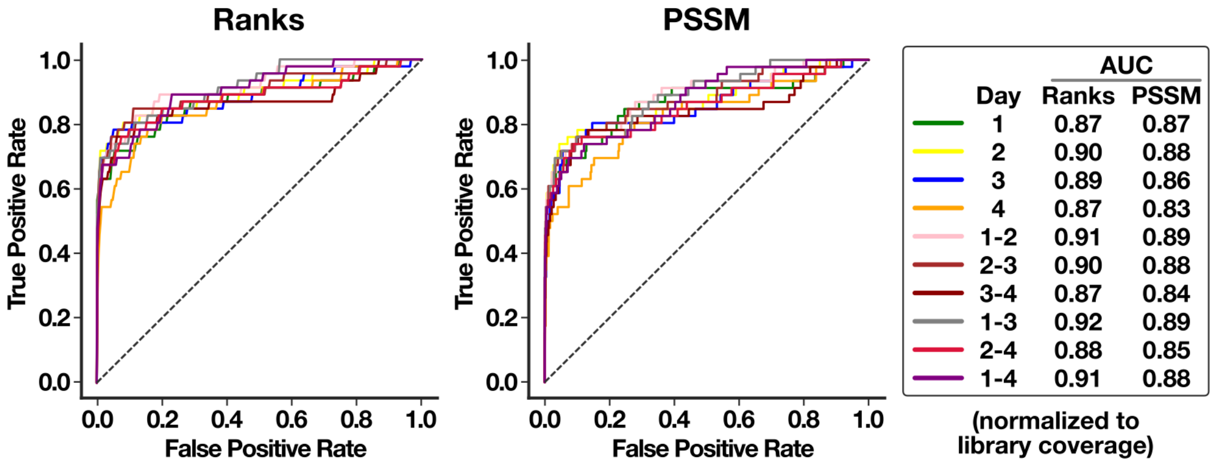

Figure S1. ROC analysis to benchmark the DMS by phage analysis PSSMs created from the by input library normalized DMS-BM selection data, compared to PSSMs generated from peptide instances curated in the ELM database.

Figure S2.

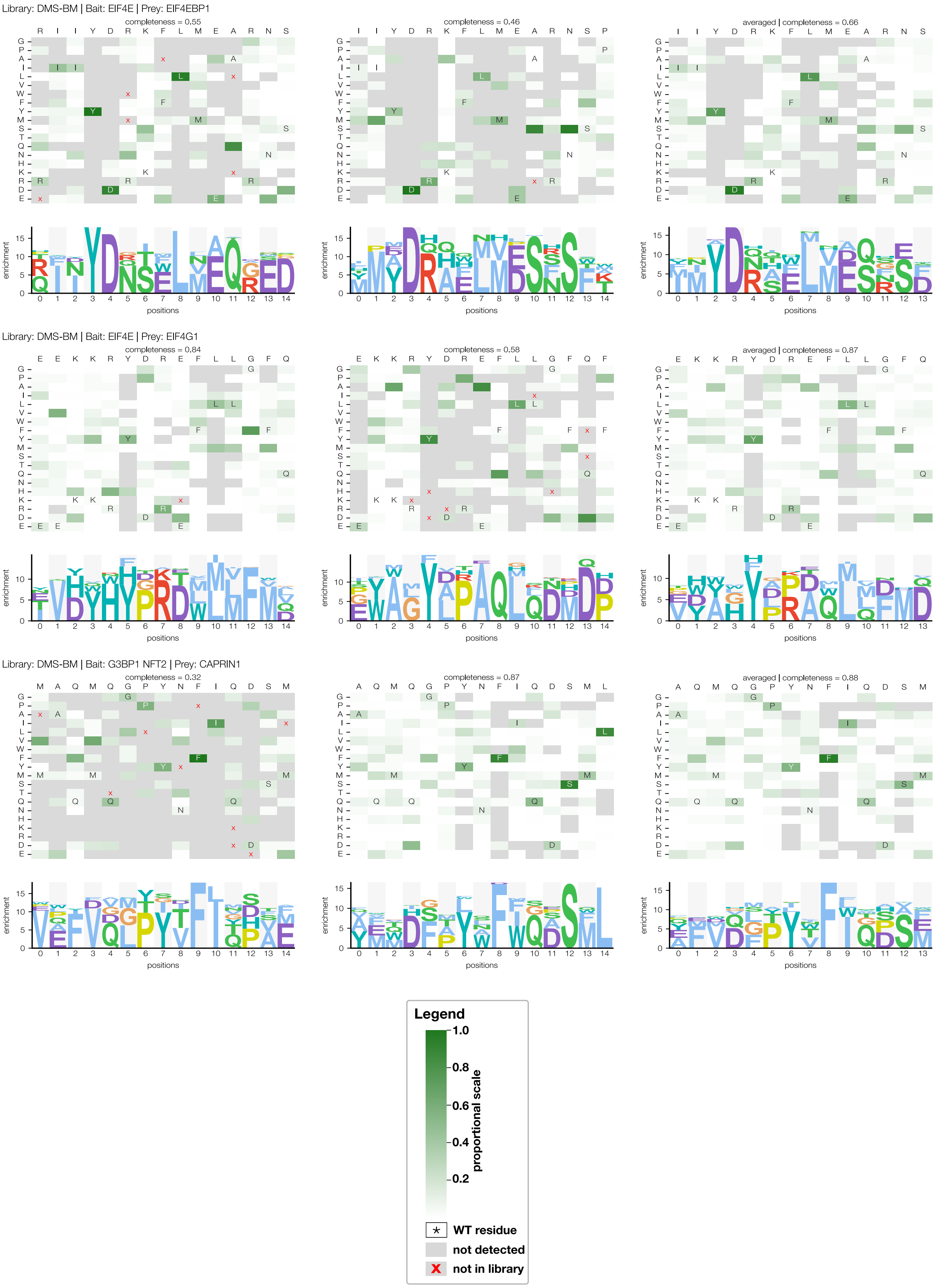
Library: DMS-BM | Bait: EIF4E | Prey: EIF4EBP1
completeness = 0.55
completeness = 0.46
averaged | completeness = 0.66
Library: DMS-BM | Bait: EIF4E | Prey: EIF4G1
completeness = 0.84
completeness = 0.58
averaged | completeness = 0.87
Library: DMS-BM | Bait: G3BP1 NFT2 | Prey: CAPRIN1
completeness = 0.32
completeness = 0.87
averaged | completeness = 0.88
enrichment
positions
Legend
proportional scale
WT residue
not detected
not in library

Fig. S2. Continued

Library: DMS-BM | Bait: G3BP1 NFT2 | Prey: USP10

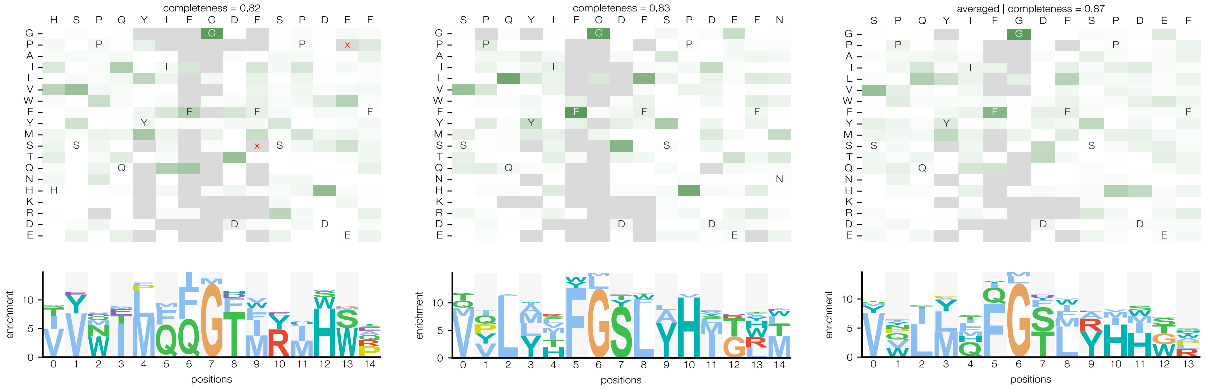

Library: DMS-BM | Bait: KEAP1 KELCH | Prey: NFE2L1

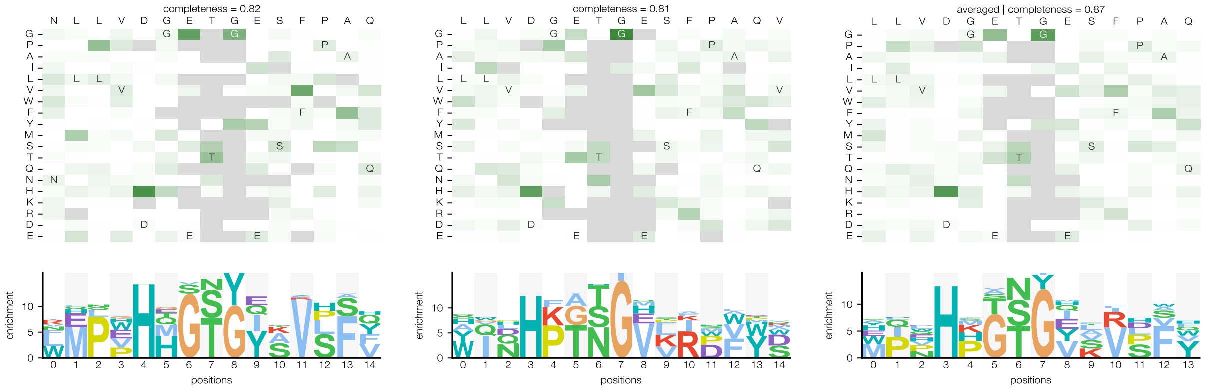

Library: DMS-BM | Bait: KEAP1 KELCH | Prey: SQSTM1

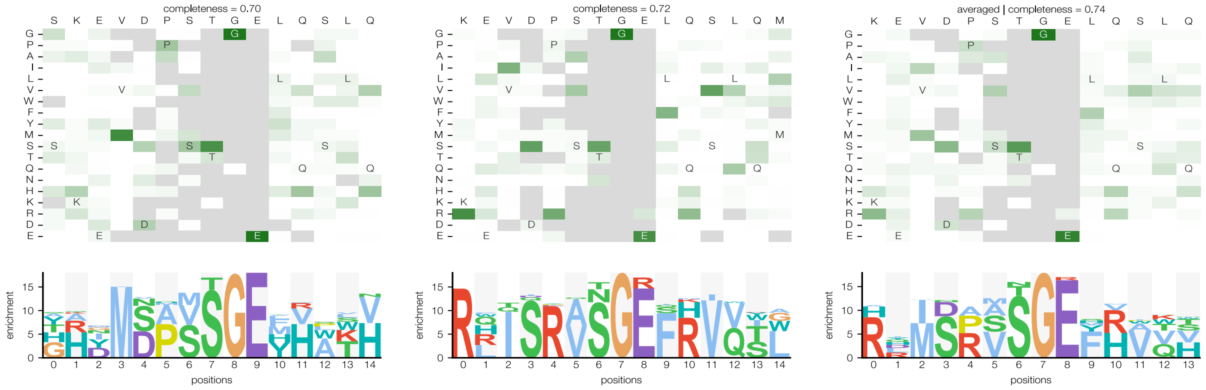

Library: DMS-BM | Bait: MDM2 SWIB | Prey: KIAA1671

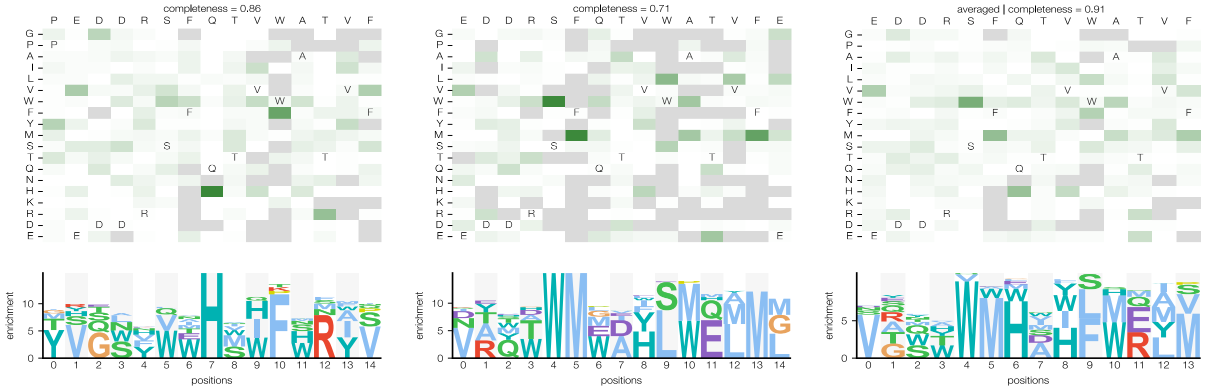

Fig. S2. Continued

Library: DMS-BM | Bait: MDM2 SWIB | Prey: RNF115

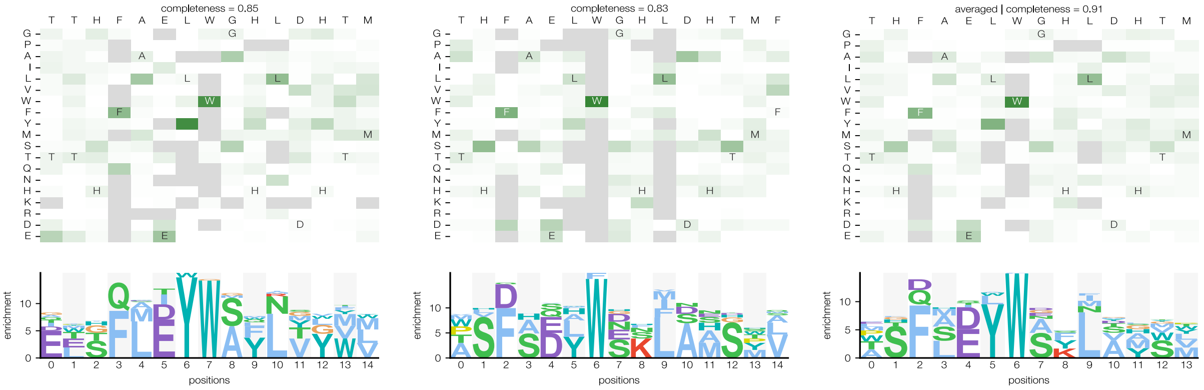

Library: DMS-BM | Bait: MDM2 SWIB | Prey: TP53

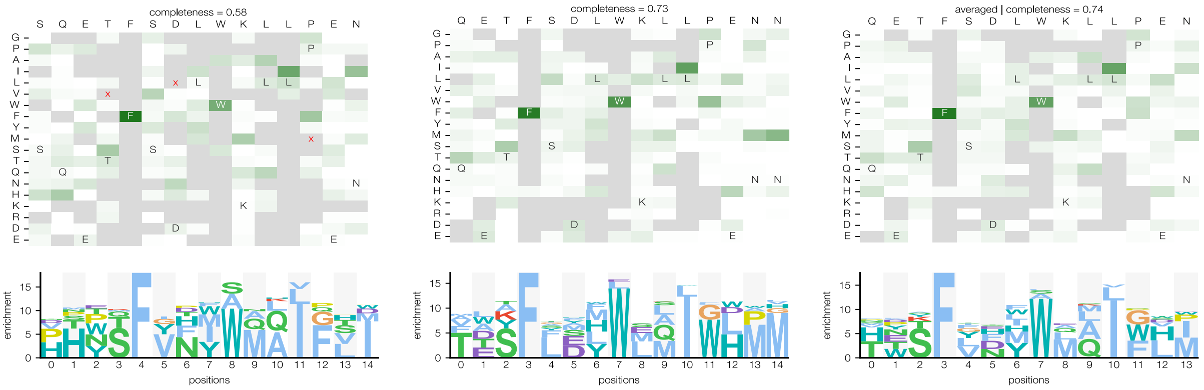

Library: DMS-BM | Bait: PABPC1 PABC | Prey: ATXN2

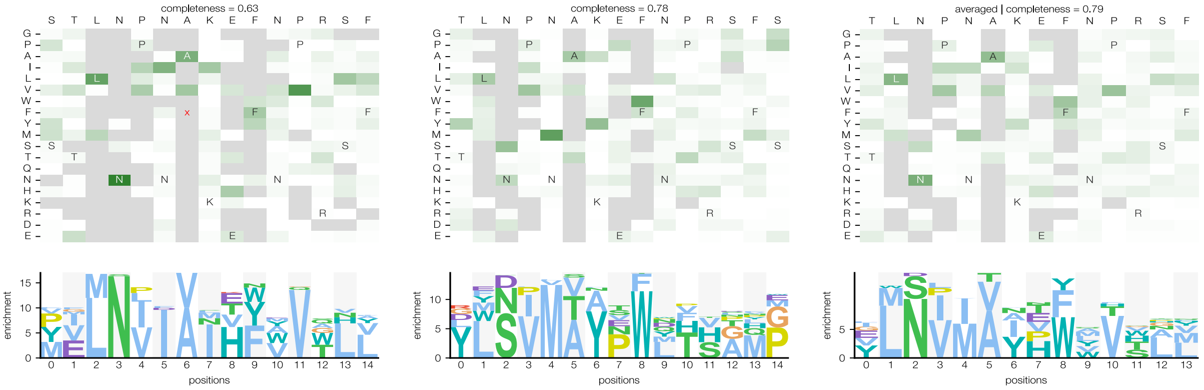

Library: DMS-BM | Bait: PABPC1 PABC | Prey: PAIP1

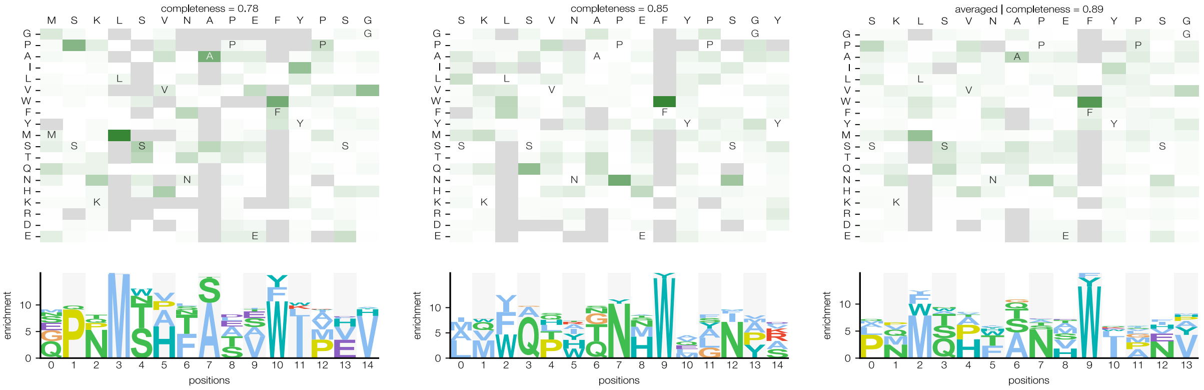

Fig. S2. Continued

Library: DMS-BM | Bait: PEX14 Pex14 | Prey: PEX5

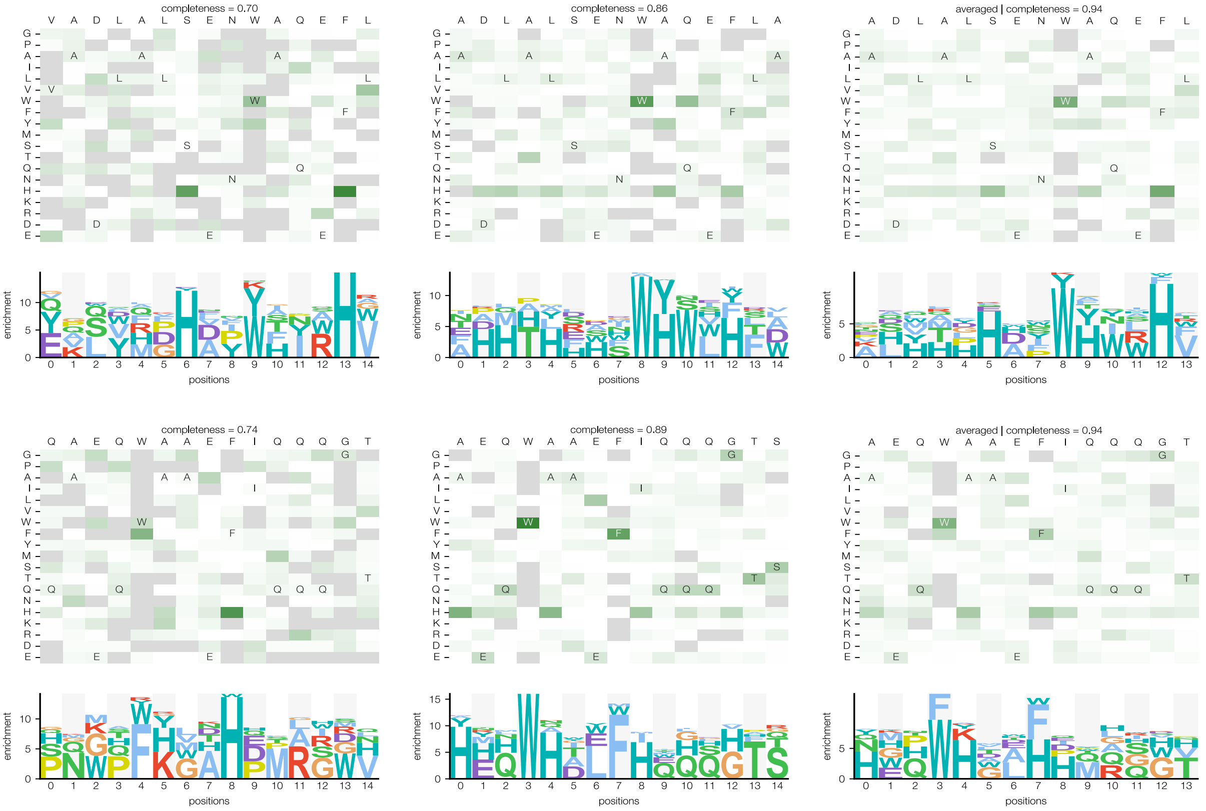

Library: DMS-BM | Bait: PP2A B56 | Prey: Axin-1

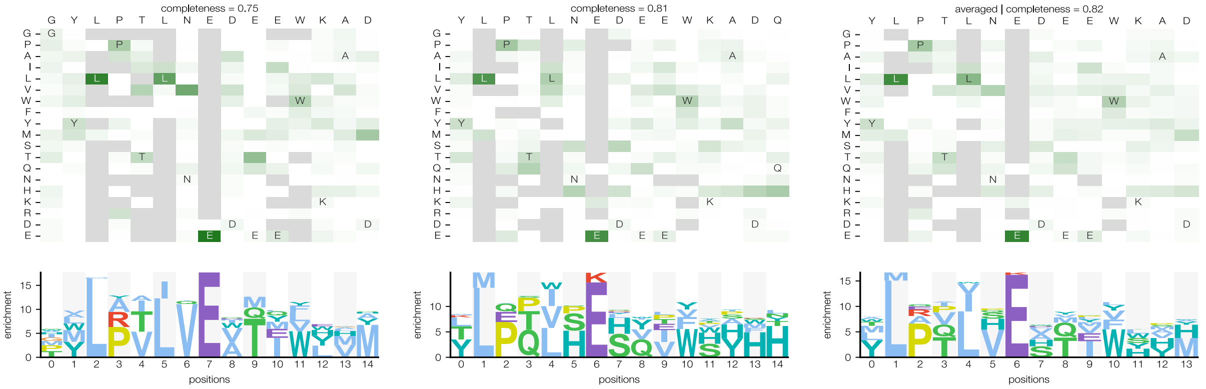

Library: DMS-BM | Bait: PP2A B56 | Prey: CDCA2

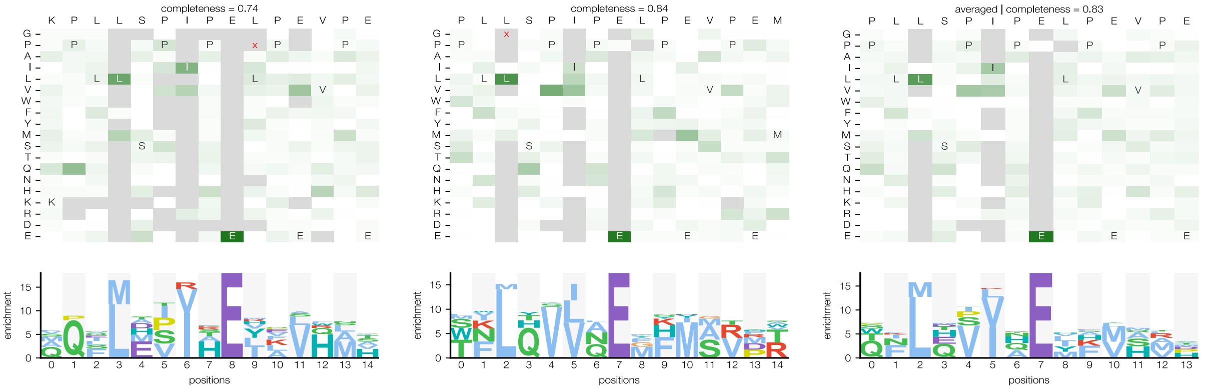

Fig. S2. Continued

Library: DMS-BM | Bait: SIN3A PAH2 | Prey: KLF9

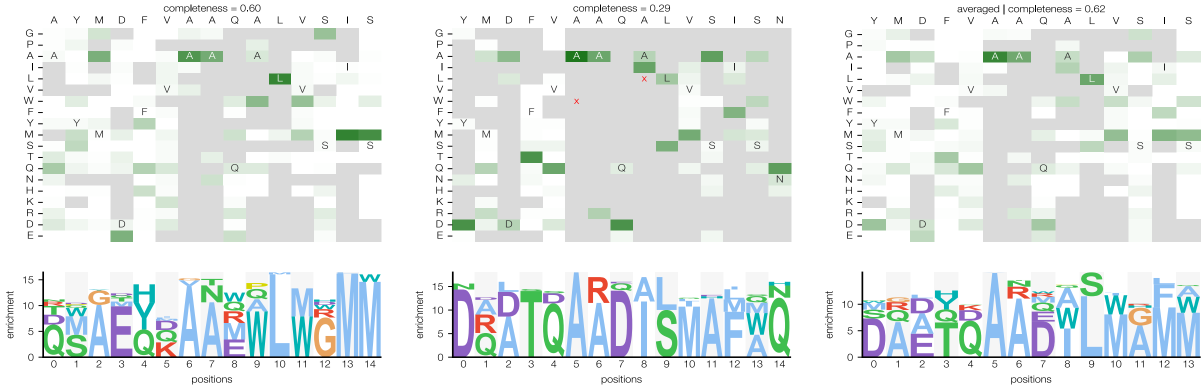

Library: DMS-BM | Bait: SIN3A PAH2 | Prey: MXI1

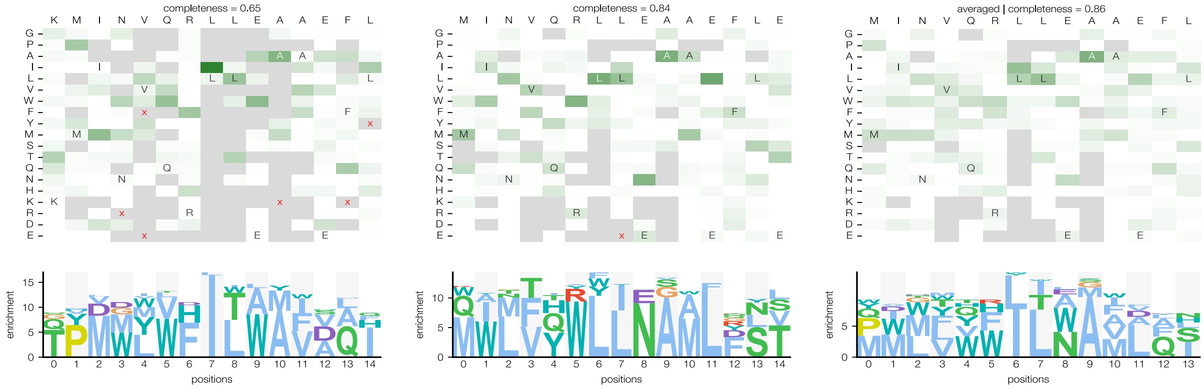

Library: DMS-BM | Bait: TLN1 PTB | Prey: PIP5K1C

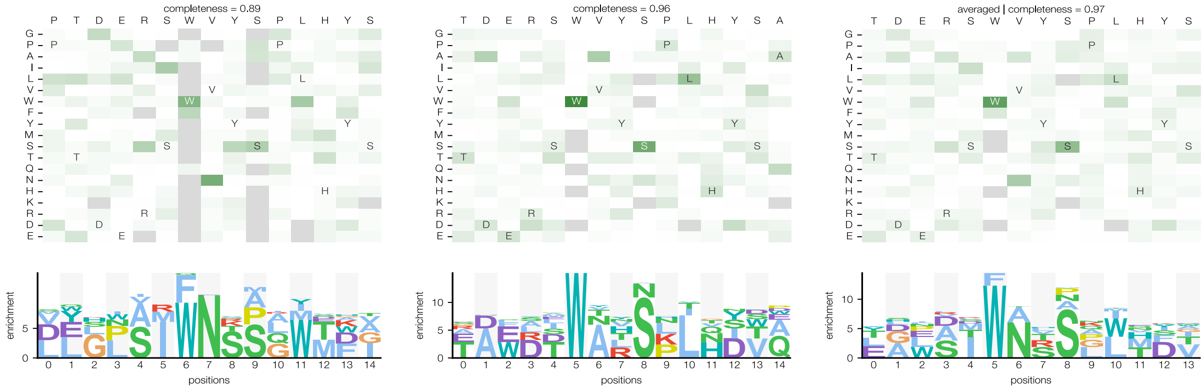

Library: DMS-BM | Bait: TLN1 PTB | Prey: TPTE2

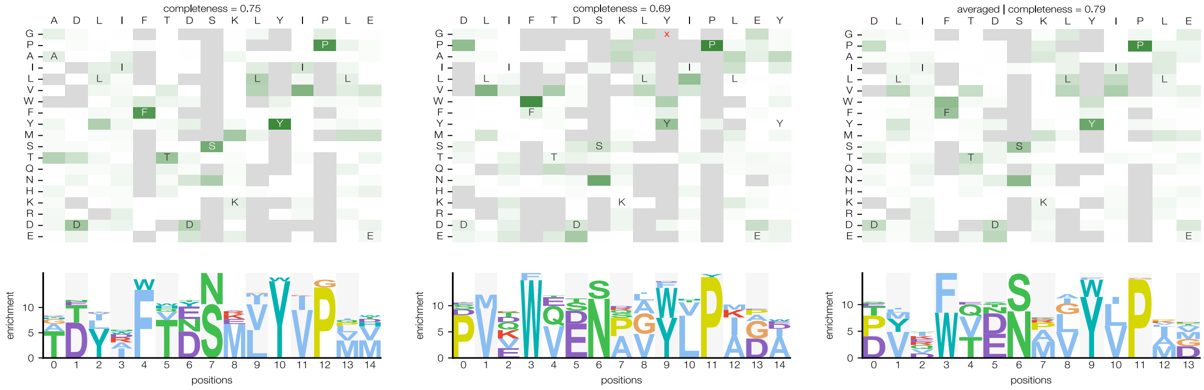

Fig. S2. Continued

Library: DMS-BM | Bait: TNKS ANK | Prey: AMOTL2

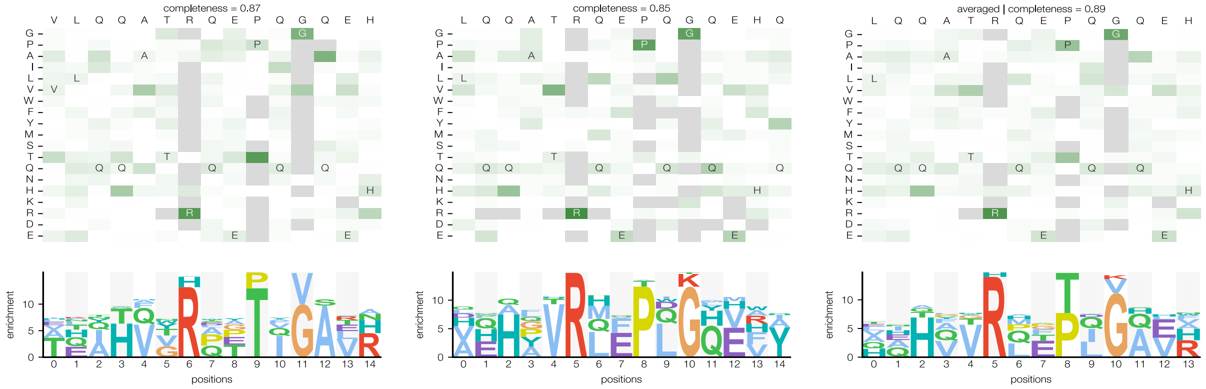

Library: DMS-BM | Bait: TNKS ANK | Prey: SH3BP2

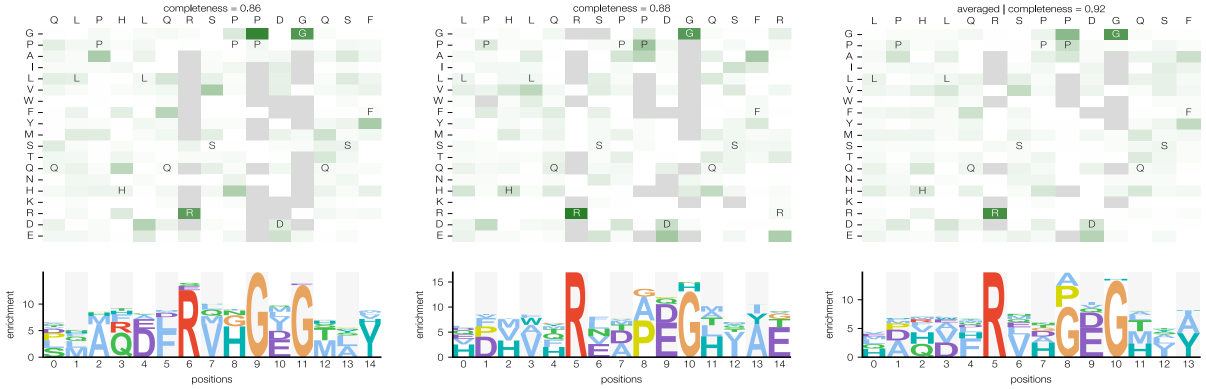

Library: DMS-BM | Bait: YES SH3 | Prey: BCAR1

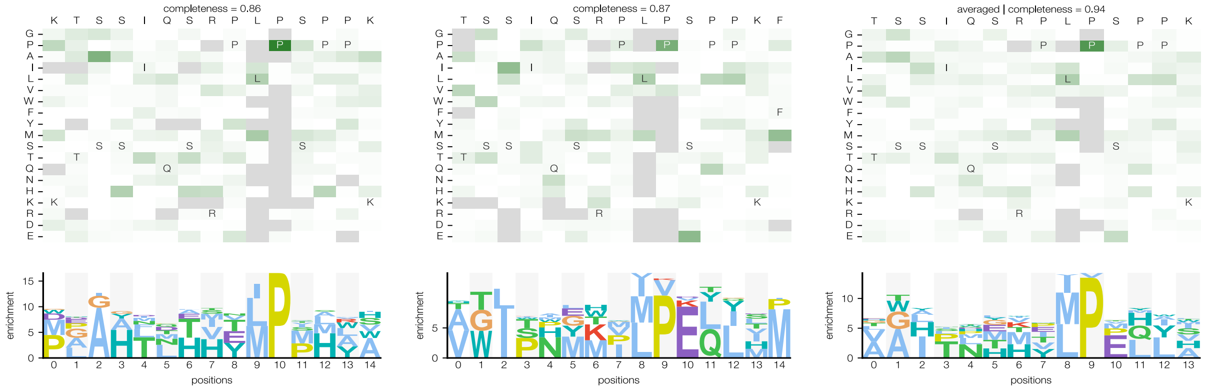

Library: DMS-BM | Bait: YES SH3 | Prey: CBL

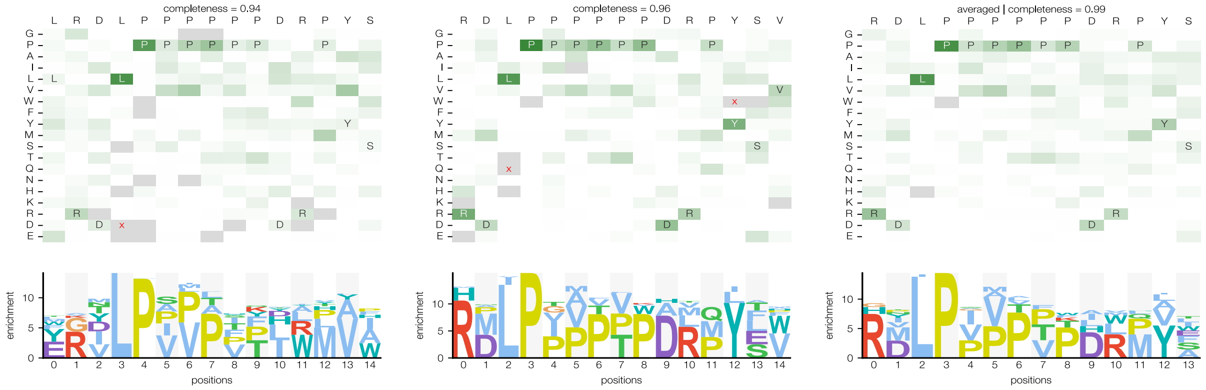

Figure S2. Heat map and PSSM representation of DMS analysis results generated through selections against the DMS-BM library. Each parental peptide is represented twice (left and middle) in overlapping registry. The averaged results are showed to the right.

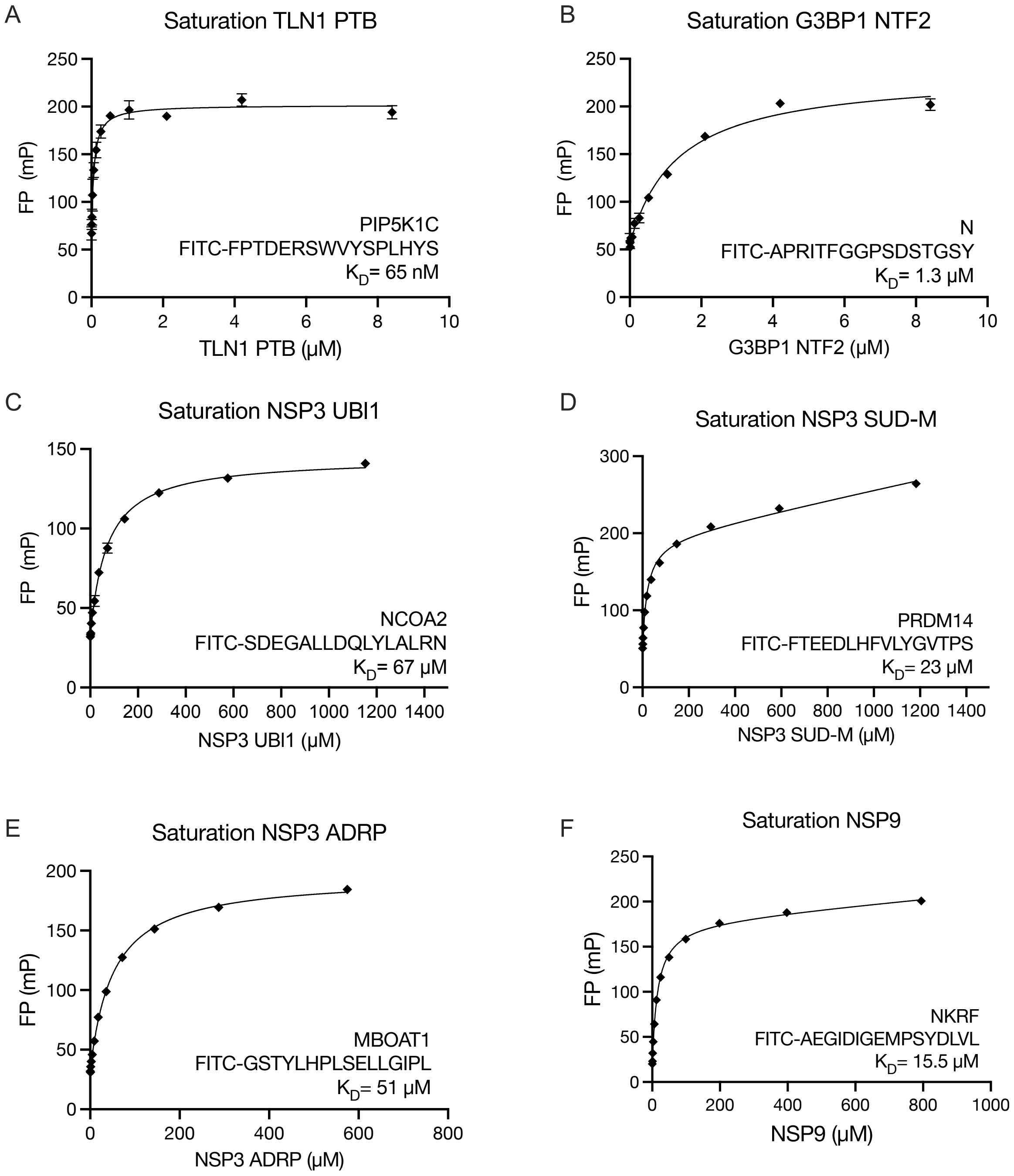

Figure S3. Binding titration curves as detected by FP of FITC-labeled probe peptides (indicated) binding to A) TLN1 PTB, B) G3BP1 NTF2, C) NSP3 UBl1, D) NSP3 SUD-M, E) NSP3 ADRP, and F) NSP9.

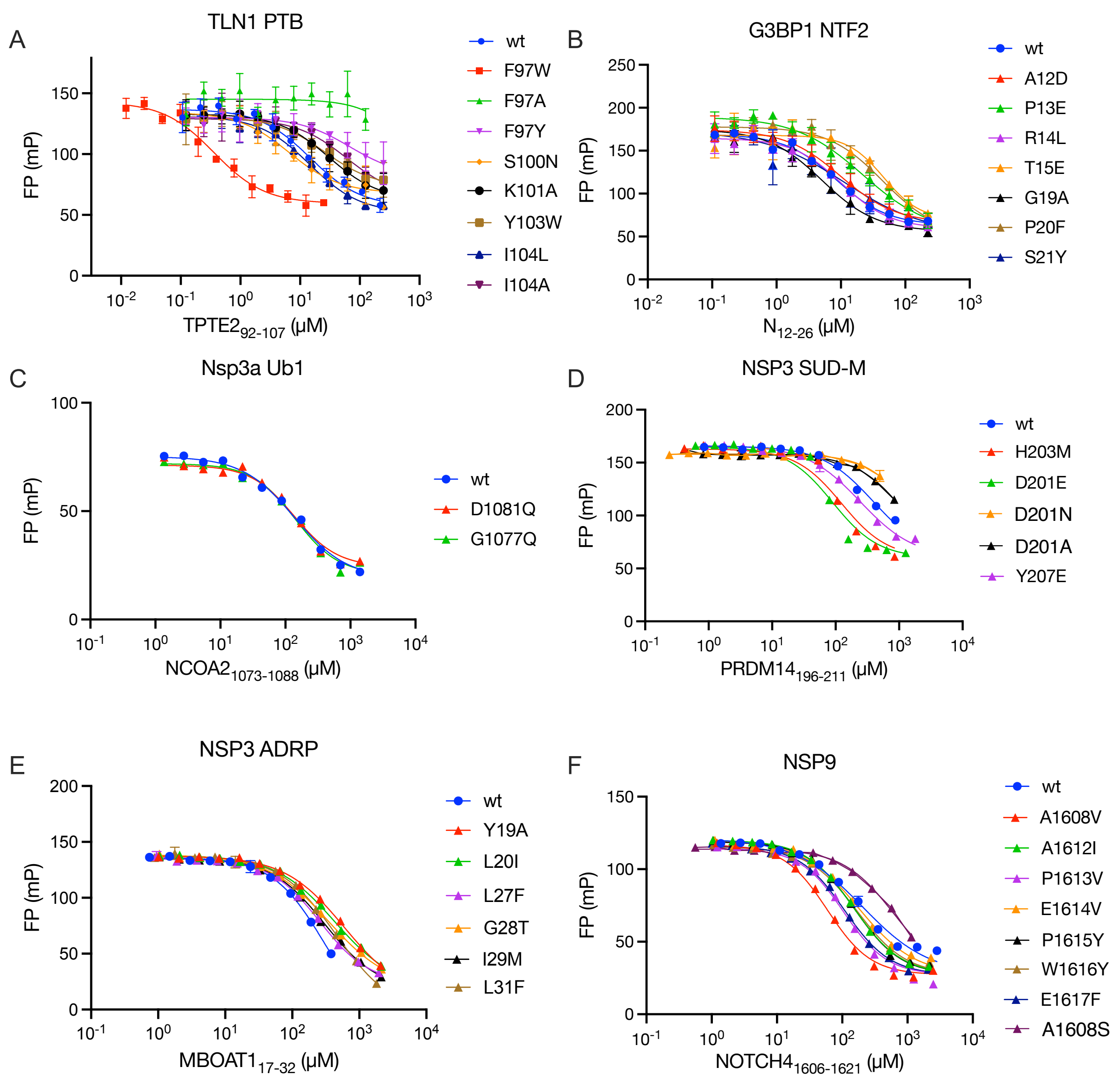

Figure S4. Affinity determinations through fluorescence polarization-based competition experiments of wild-type and mutant TLN1 PTB, B) G3BP1 NTF2, C) NSP3 UBI1, D) NSP3 SUD-M, E) NSP3 ADRP, and F) NSP9.

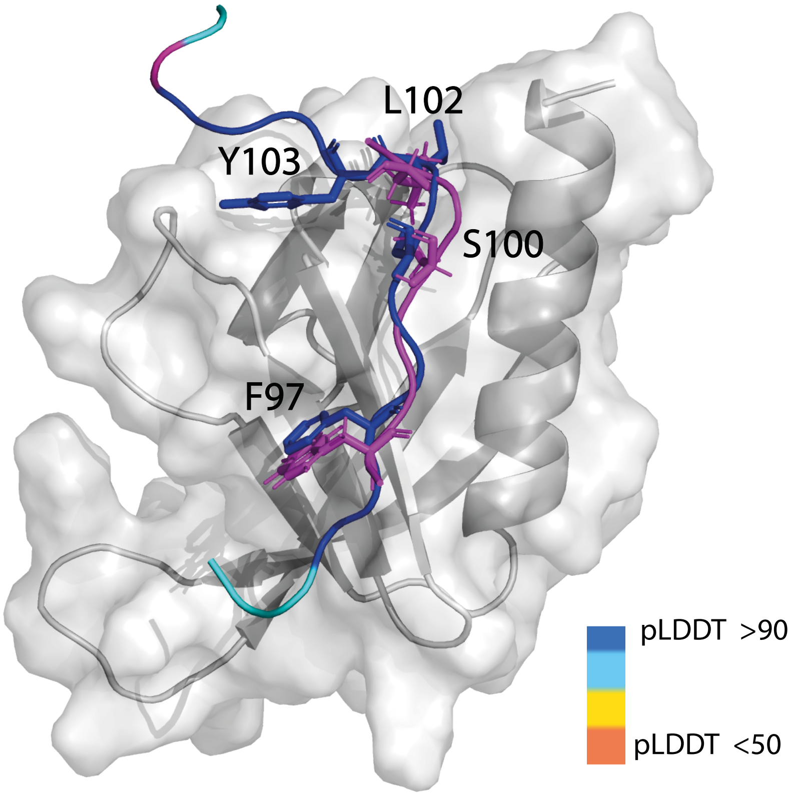

Figure S5. AlphaFold3 model of the TLN1 PTB-TPTE2 complex overlayed with the previously solved NMR structure of TLN1 PTB in complex with PIP5K1C (PDB id 2G35; peptide in magenta). The TPTE2 peptide is colored according to the plDDT score, which shows the high confidence of the model (dark blue very high confidence plDDT > 90; light blue plDDT 90 > plDDT > 70). The figure is a larger version of Fig. 2K.

Figure S6.

Library: DMS-CoV | Bait: ABL2 SH3 | Prey: N

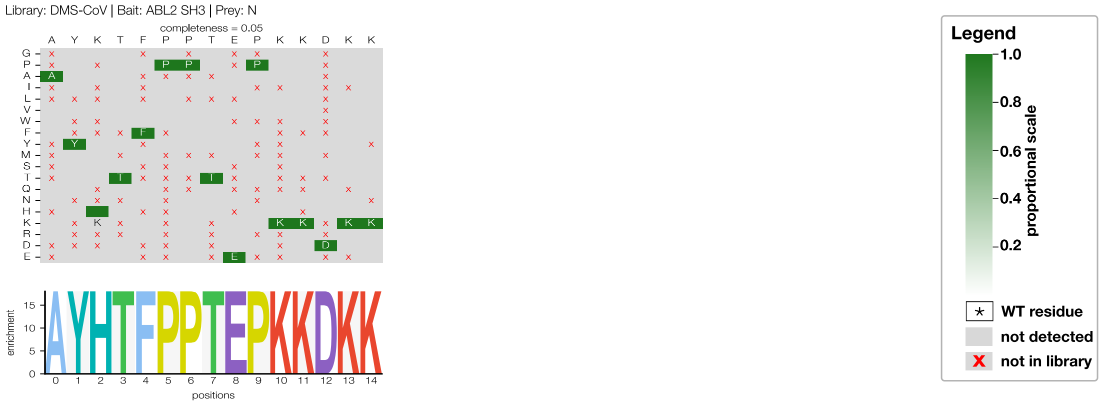

Library: DMS-CoV | Bait: AP2M1 MU | Prey: NSP14

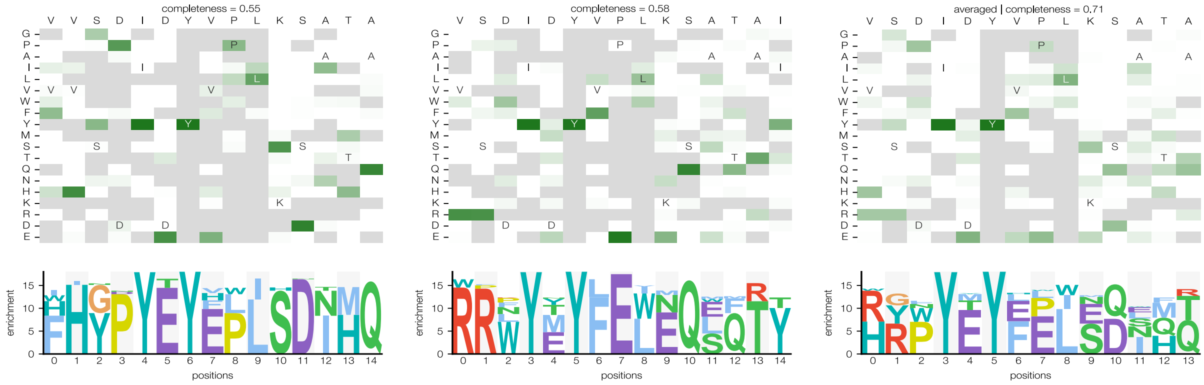

Library: DMS-CoV | Bait: EZR FERM | Prey: E

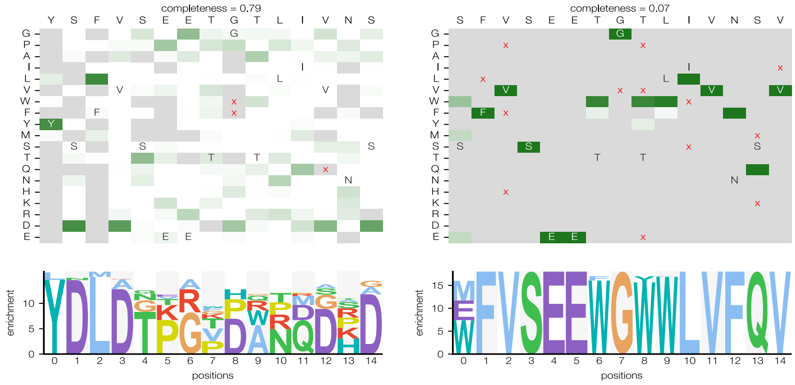

Library: DMS-CoV | Bait: G3BP1 NFT2 | Prey: N

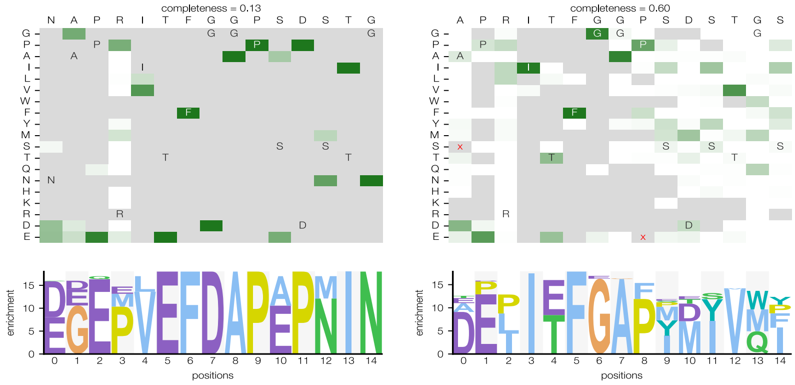

Figure S6. Continued.

Library: DMS-CoV | Bait: G3BP1 NFT2 | Prey: NSP3

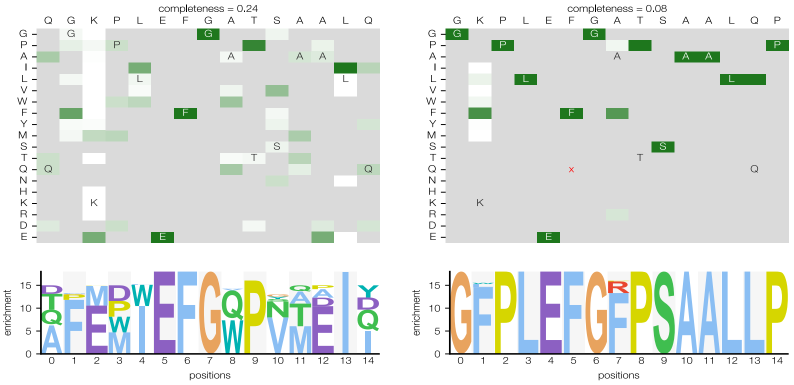

Library: DMS-CoV | Bait: NSP16 | Prey: DYRK1B

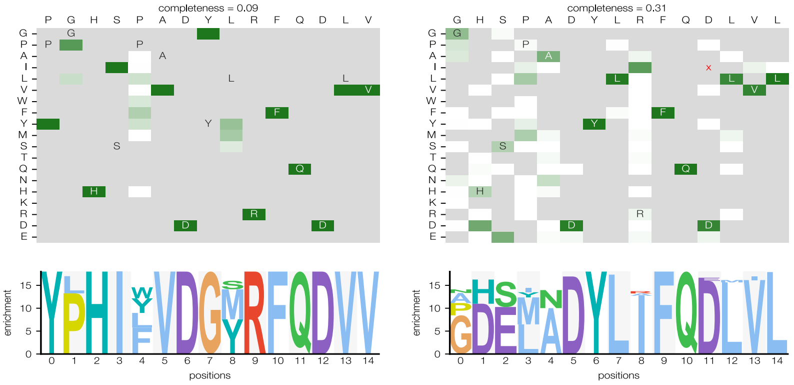

Library: DMS-CoV | Bait: NSP16 | Prey: ICA1L

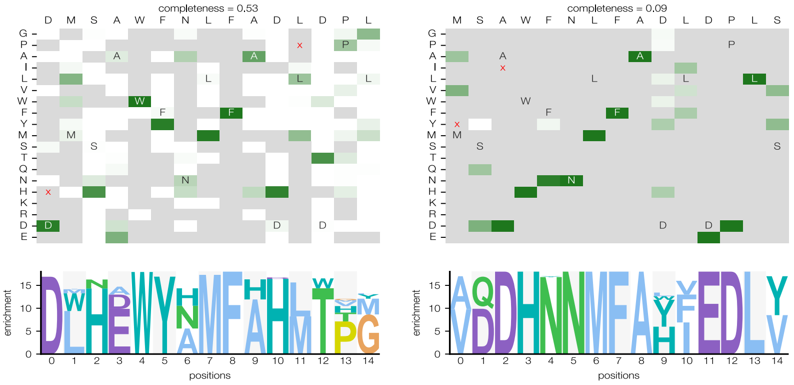

Library: DMS-CoV | Bait: NSP3 ADRP | Prey: AZIN2

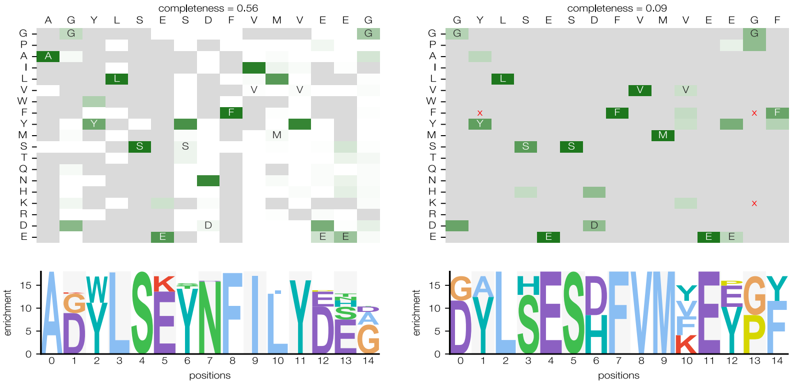

Figure S6. Continued.

Library: DMS-CoV | Bait: NSP3 ADRP | Prey: MBOAT1

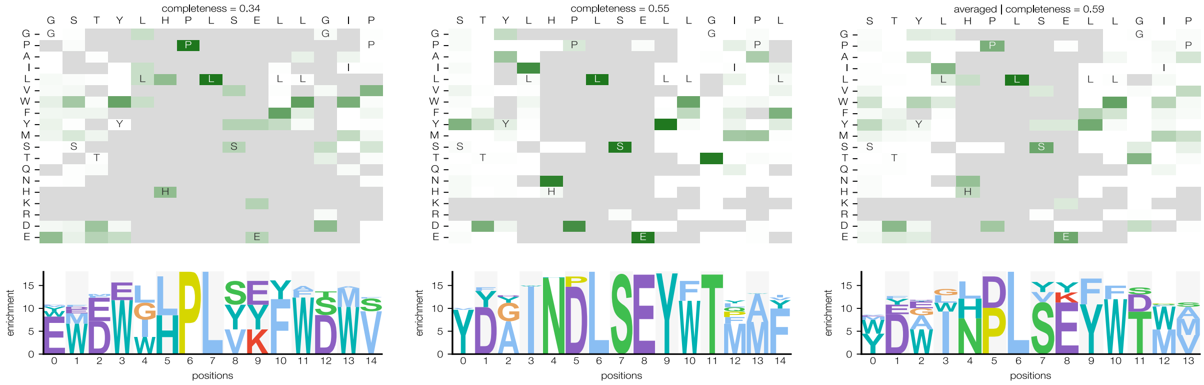

Library: DMS-CoV | Bait: NSP3 SUD-M | Prey: PRDM14

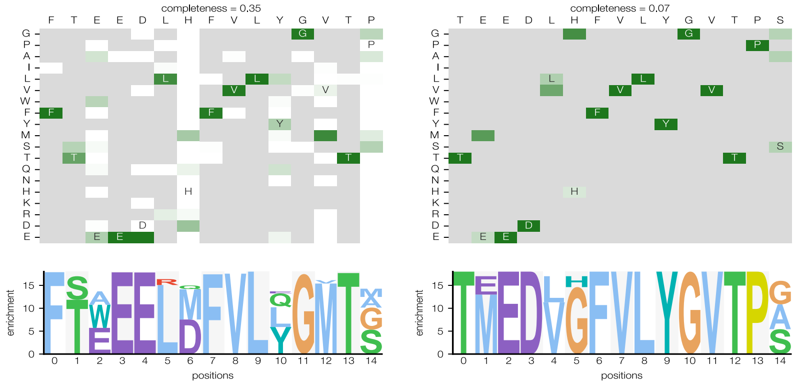

Library: DMS-CoV | Bait: NSP3 SUD-M | Prey: TET3

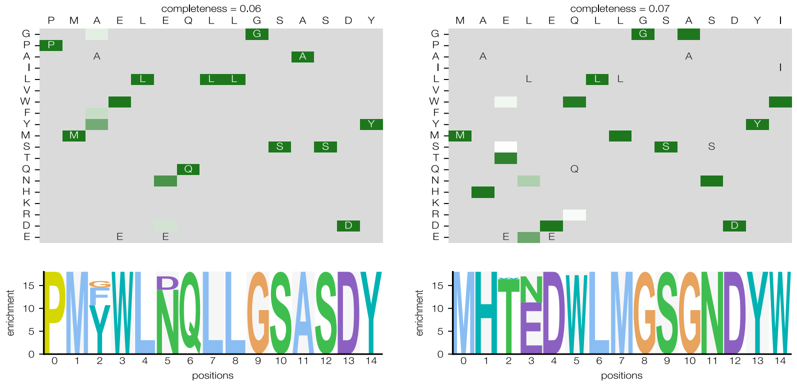

Library: DMS-CoV | Bait: NSP3 UBI1 | Prey: NCOA2

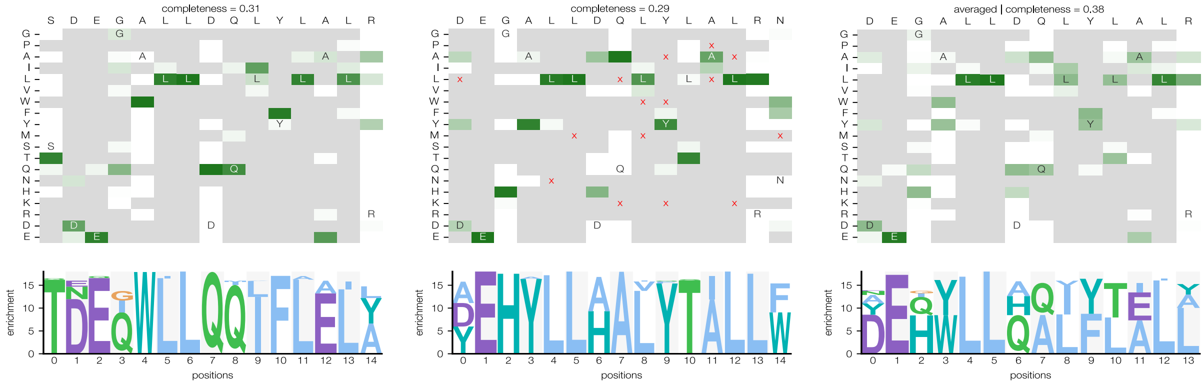

Figure S6. Continued.

Library: DMS-CoV | Bait: NSP3 UBl1 | Prey: NYNRIN

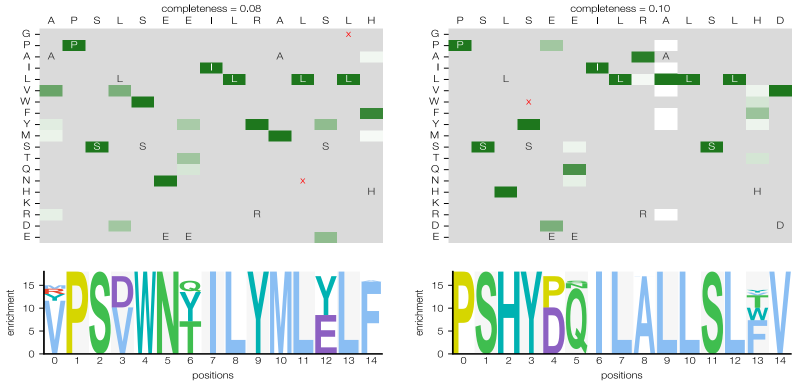

Library: DMS-CoV | Bait: NSP9 | Prey: AXIN1

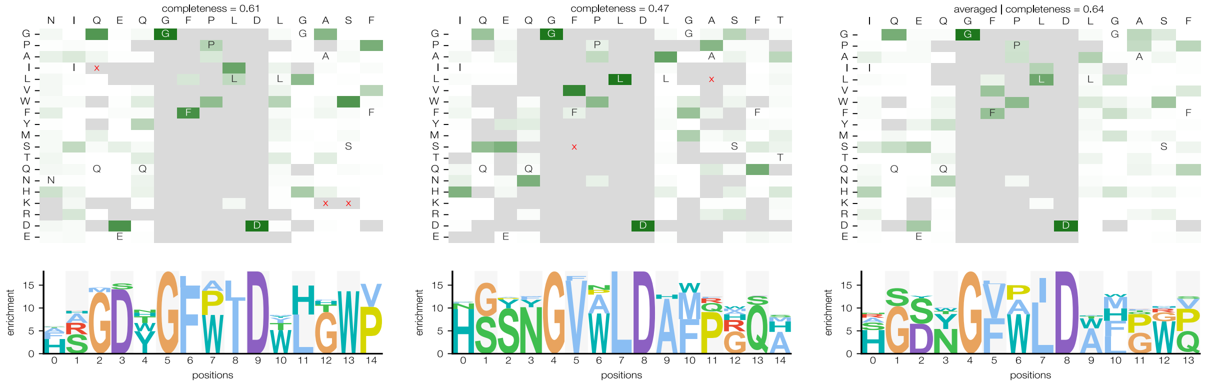

Library: DMS-CoV | Bait: NSP9 | Prey: NEK9

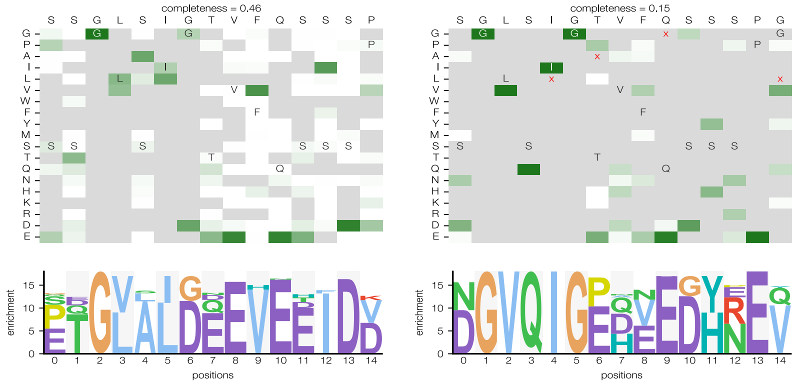

Library: DMS-CoV | Bait: NSP9 | Prey: NOTCH4

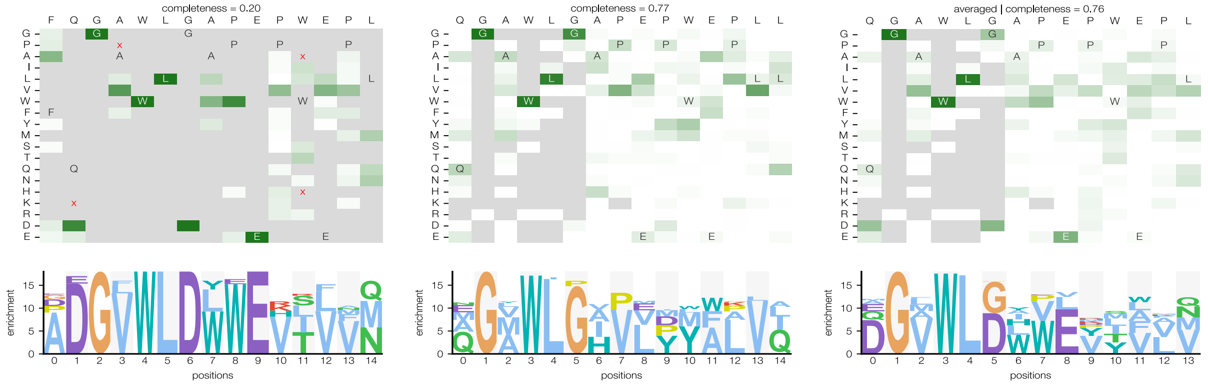

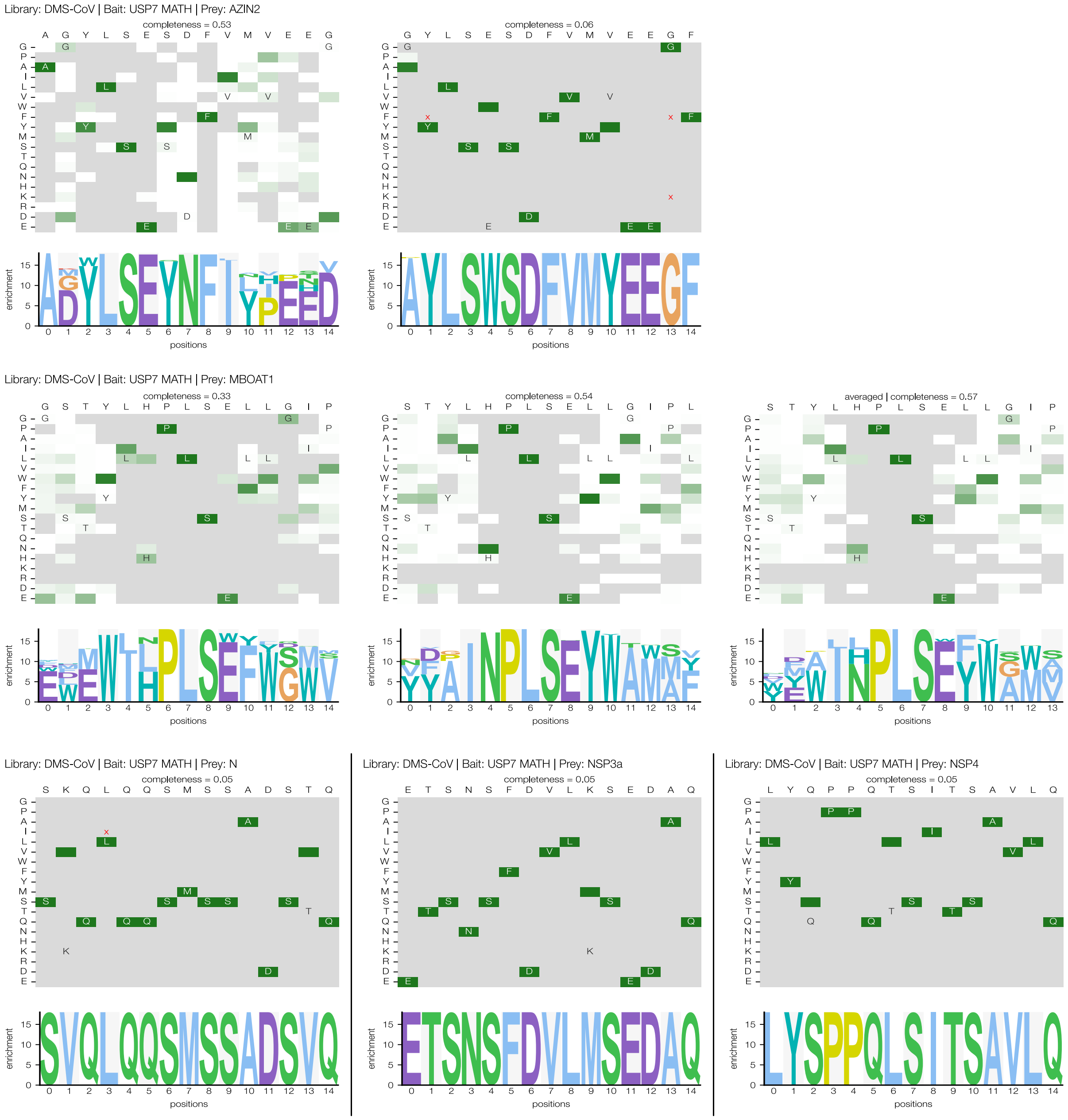

Figure S6. Heat map and PSSM representation of DMS analysis results generated through selections against the DMS-CoV library. Each parental peptide is represented twice (left and middle) in overlapping registry. The averaged results are showed to the right when available.

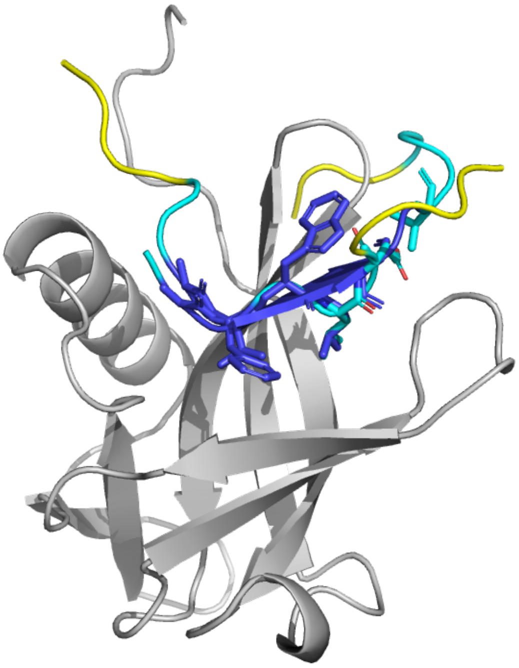

Figure S7. AlphaFold3 model of the complex of NSP9 and the NOTCH4 A1603V/P1613V peptide (QGVWLGAVEPWEPL) overlayed with the model of the AXIN (IQEQGFGFPLDLGAS). Peptide coloring is according to the pLLDT score (deep blue = high confidence). Generated using PyMol.

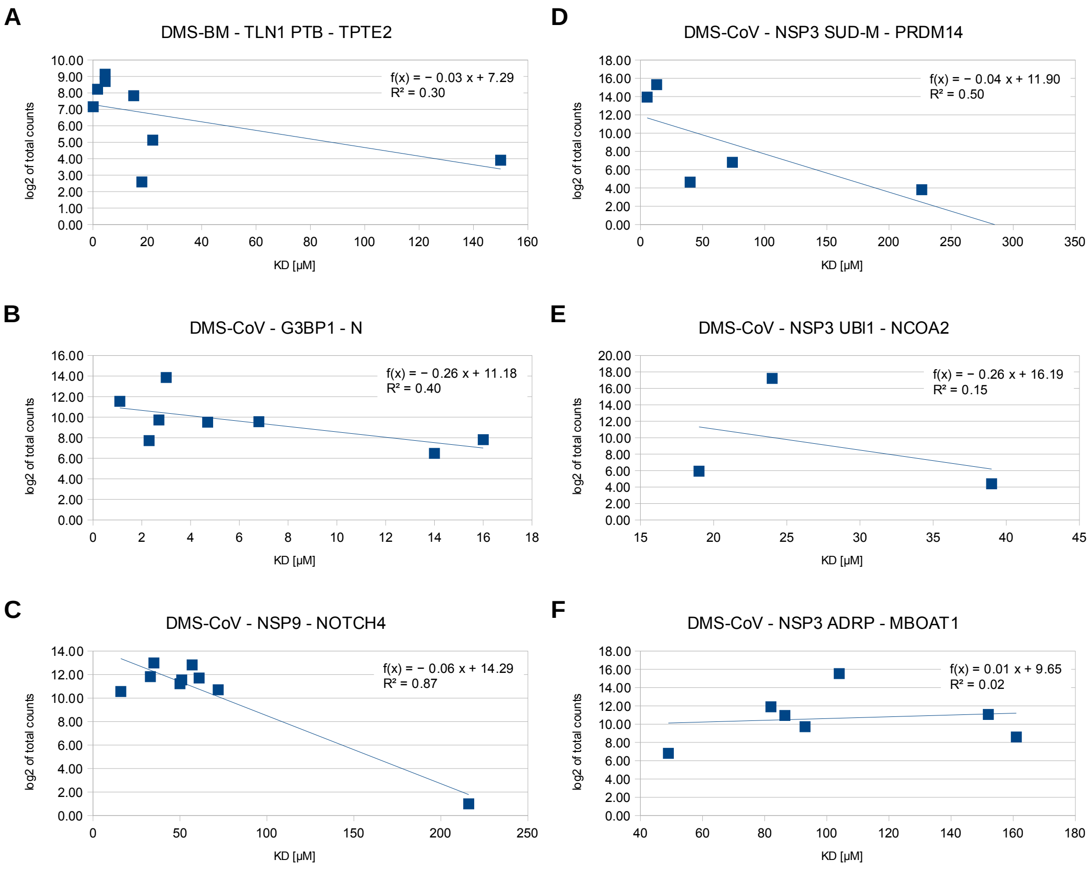

Figure S8. Correlation between NGS counts and affinities. The log2 of the total NGS counts of a peptide variant observed in selection against a given bait plotted against the measured affinities. Data is shown for (A) TLN1 PTB domain and the TPTE2$_{92-107}$ peptide, (B) G3BP1 NTF and the N$_{12-26}$ peptide, (C) NSP9 and the NOTCH4$_{1606-1621}$ peptide, (D) NSP3 SUD-M and the PRDM14$_{196-211}$ peptide, (E) NSP3 UBl and the NCOA2$_{1073-1088}$ peptide, and (F) NSP3 and the MBOAT1$_{17-32}$ peptide.
